# Alzheimer’s disease detection from structural brain MRI using FFA U-Net segmentation and transfer learning

**DOI:** 10.3389/fnins.2026.1871982

**Published:** 2026-09-03

**Authors:** Nirupama Panabakam, Karthik Elangovan, Koteeswaran Seerangan, Nalini Manogaran, Karthiga Malleeswaran, Sumendra Yogarayan

**Affiliations:** 1 Department of Computer Science and Engineering, VEMU Institute of Technology, Chitoor, Andhra Pradesh, India; 2 Department of Computer Science and Engineering, School of Computing, Vel Tech Rangarajan Dr. Sagunthala R&D Institute of Science and Technology, Avadi, Chennai, Tamil Nadu, India; 3 Department of Computer Science and Engineering, R. M. K. Engineering College (Autonomous), Kavaraipettai, Tamil Nadu, India; 4 Department of Computer Science and Engineering (Cyber Security), S.A. Engineering College (Autonomous), Chennai, Tamil Nadu, India; 5 Department of Computer Science and Engineering, Bannari Amman Institute of Technology, Sathyamangalam, Tamil Nadu, India; 6 Faculty of Information Science and Technology (FIST), Multimedia University (MMU), Melaka, Malaysia

**Keywords:** ADNI, Alzheimer’s disease detection, brain MRI segmentation, feature fusion attention, FFA U-Net, OASIS, transfer learning, VGG16

## Abstract

**Introduction:**

Alzheimer’s disease (AD) is a progressive neurodegenerative disorder that leads to a gradual decline in cognitive and memory function. Multimodal neuroimaging, particularly magnetic resonance imaging (MRI), has become a central diagnostic tool for tracking disease progression, supporting diagnosis, treatment planning, and follow-up monitoring. Manual delineation of brain structures by clinicians remains time-consuming and labor-intensive, and computer-assisted segmentation is complicated by spatial and structural variability as well as intensity inhomogeneity across images. This study proposes an integrated framework for automated brain image segmentation and AD classification to address these challenges.

**Methods:**

The framework combines a modified U-Net architecture, termed FFA U-Net, with a fine-tuned VGG16 classifier. FFA U-Net incorporates a residual inception module and a feature fusion attention mechanism into the standard U-Net backbone to perform end-to-end brain tissue segmentation. Segmented outputs are subsequently passed to the fine-tuned VGG16 model for classification of AD status. The framework was evaluated on two datasets, ADNI (843 images) and OASIS (416 images), using subject-wise, non-overlapping training, validation, and test splits to prevent data leakage.

**Results:**

The proposed framework achieved a segmentation Dice Similarity Coefficient (DSC) of 0.929 and a classification accuracy of 98.90%, based on a single data split. These results were consistent across both the ADNI and OASIS datasets, indicating competitive segmentation and classification performance.

**Discussion:**

The findings suggest that combining attention-augmented segmentation with transfer learning-based classification can effectively support automated AD detection from structural MRI, potentially reducing the manual burden on clinicians. As results are based on a single split, further validation using cross-validation or independent cohorts is warranted to confirm robustness and generalizability. The framework should currently be regarded as an experimental research tool that requires additional clinical validation before deployment in practice.

## 1 Introduction

Alzheimer disease (AD) is a progressive neurodegenerative disease and the most common type of dementia in the global population with an approximated 60 to 80 percent of all the cases of dementia worldwide. It is a progressive decline in cognitive abilities, such as memory, thinking, and the regulation of behaviour, in older adults aged 65 years or older (Zhang et al., 2015). With the world population on the verge of aging, the burden of AD is bound to increase manifold, making the early and accurate diagnosis of AD a critical issue in terms of clinical intervention and care planning.

Traditional diagnosis of AD refers mainly to post-symptomic neuropsychological assessment, such as the Mini-Mental State Examination (MMSE), Clinical Dementia Rating (CDR) and neuropsychiatric questionnaires (Salvatore et al., 2014). These tests are complemented with biomarker tests, cerebral spinal fluid (CSF) profiling and neuroimaging modalities, which are Magnetic Resonance Imaging (MRI) and Positron Emission Tomography (PET). Nevertheless, as the vast majority of people become asymptomatic until the disease has reached an advanced stage, the approaches based on symptoms are inherently limited in the early-stage detection (Malcolm and De Santi, 2023). It highlights the importance of automated, imaging, diagnostic tools that can detect subtle structural changes in the brain at earlier stages.

The computer aided diagnosis based on MRI has come out as a promising approach in the detection of AD due to its ability to provide detailed structural information about brain atrophy, loss of grey matter and expansion of cerebrospinal fluid, which are hallmarks of AD progression. Conventional methods of machine learning, including Support Vector Machines (SVM) in combination with hand-crafted extraction of features in high-dimensional MRI data have been extensively used to classify AD (De Bruijne, 2016; Rathore et al., 2017). Although useful to some extent, these approaches are labour-intensive, need domain knowledge to engineer features, and do not generalize well across different imaging datasets.

Deep learning, specifically Convolutional Neural Networks (CNNs) have revolutionized the analysis of medical images by allowing hierarchical, end-to-end learning of features directly off raw image data without manual intervention (Rathore et al., 2017). They are also suitable in the detection of the delicate, spatially dispersed patterns that are related to AD (Manikandan et al., 2023). Based on this, multimodal techniques that integrate MRI and PET with Stacked Autoencoders (SAEs) have been shown to perform better with AD classification than either unimodal methods or the more computationally intensive multimodal methods (Shi et al., 2017). The longitudinal studies that have used Recurrent Neural Networks (RNNs) and Multi-Layer Perceptrons (MLPs) to conduct temporal analyses of MRI have achieved classification accuracies of about 89.69 percent when differentiating AD and normal controls (Cui et al., 2018). More recently, ensemble Deep CNN (DCNN) models combining predictions of multiple models have further enhanced classification accuracy in multi-class AD staging tasks (AlSaeed and Omar, 2022).

Although this has been done, one of the main limitations with many of the existing methods is the lack of a formal step of brain tissue segmentation before classification. In the absence of structured segmentation, the classifiers are frequently subjected to background noise, scanner related artefacts and anatomically irrelevant regions, which reduces the accuracy and interpretability. More so, standard U-Net architectures employed in segmentation do not necessarily prioritize the most discriminative spatial areas or feature channels, which restrict their usefulness in the analysis of AD-specific MRI.

To overcome these obstacles, this paper recommends a two-step deep learning model to perform automated AD detection. A refined U-Net architecture, called FFA U-Net, is used in the first stage to perform pixel-wise brain tissue segmentation. FFA U-Net adds a residual inception module to the encoder-decoder backbone to allow multi-scale feature extraction with less computational cost, and adds a Feature Fusion Attention (FFA) module within the skip connections to adaptively emphasize discriminative spatial regions and informative feature channels. The binary tissue probability maps generated by FFA U-Net are then merged with the original MRI slices and sent into a fine-tuned VGG16 network to effectively classify and detect AD in the second stage. The main contributions of this work are as follows. In contrast to standard two-stage pipelines that segment the image to crop or mask a region of interest for classification, our approach also passes structured grey-matter, white-matter and cerebrospinal-fluid tissue-probability maps as anatomical prior information to the classifier, which uses a biologically meaningful structure for classification. There are three novelties. First, the U-Net skip connections are processed in parallel in both spatial and channel dimensions, then the two are fused together with a parameter-free residual additive fusion. Unlike previous segmentation-attention approaches (Table 1), these were applied in a non-sequential manner and not just at the network “bottleneck.” Second, the limitation of the fixed-receptive-field of common variants of U-Net is mitigated by replacing the plain convolutions with residual inception blocks for multi-scale tissue-boundary extraction. Third, the fine tuning of VGG16 is partial, only the last convolutional layer is fine-tuned while the entire network, or a randomly sampled subset of layers, is not. To the best of our knowledge, such an anatomically structured segmentation input, parallel dual attention (with additive fusion) and selective fine-tuning approach has not been reported in combination for AD detection.

**Table 1:** Summary and comparison of representative related studies on Deep Learning-based AD detection.

Reference	Year	Method	Dataset	Segmentation	Attention	Key Metric	Limitation
Wen et al. (2020)	2020	CNN (multiple architectures)	ADNI	No	No	Reproducibility analysis	Data leakage risk in prior works
Liu et al. (2021)	2021	Depthwise Separable CNN	ADNI	No	No	Competitive accuracy	No attention mechanism
Khan et al. (2023)	2023	VGG16/VGG19 Transfer Learning	ADNI	GM extraction	No	Multi-class staging	Overfitting on small datasets
Tabish et al. (2025)	2025	VGG16, DenseNet, ResNet (comparative)	ADNI	No	No	VGG16: 95.0%	ResNet50 underperforms (62%)
Jin et al. (2024)	2024	3D DenseNet + MobileNetV3	ADNI	✅ 3D	CA + GAM	3-class classification	High computational cost
Sorour et al. (2024)	2024	Systematic review (3D DL)	ADNI/OASIS	✅ Reviewed	Reviewed	—	No novel architecture proposed
Alp et al. (2025)	2025	ViT + Time-Series Transformer	ADNI	No	Self-attention	99.04% (binary)	Large data dependency
Castro-Silva et al. (2024)	2024	3D ViT (MIMD-3DVT)	ADNI/OASIS/AIBL	ROI-based	Self-attention	97.14%	Multi-dataset complexity
Shaffi et al. (2024)	2024	Ensemble of ViTs	ADNI/OASIS	No	Self-attention	~2% gain over single ViT	High compute cost
Wong et al. (2024)	2024	GAN + CNN	OASIS	No	No	Data-efficient accuracy	GAN distribution shift risk
Proposed FFA U-Net	2024	FFA U-Net + Fine-tuned VGG16	ADNI + OASIS	Pixel-wise	Spatial + Channel (FFA)	98.90% / DSC 0.929	Moderate compute increase

The rest of this paper will be structured as follows. Section 2 discusses related research in the field of deep learning-based AD detection. Section 3 describes the proposed methodology in detail. Section 4 provides the experimental set up, findings and discussion. Section 5 will be the conclusion of the paper, which will summarize the findings and direction of future research.

## 2 Related works

### 2.1 CNN-based approaches for AD classification

Convolutional Neural Networks (CNNs) have been the most effective tool to be used in Alzheimer disease (AD) detection using deep learning since it has been shown to be superior to traditional machine learning methods in extracting hierarchical features directly out of MRI data. Wen et al. (2020) presented a detailed reproducibility-oriented assessment of CNN architectures to classify AD, and demonstrated that the quality of model performance is highly sensitive to data leakage and data partitioning strategies and preprocessing choices. Based on the conventional CNN design, Liu et al. (2021) introduced depthwise separable convolutions to reduce the computational load and keep the classification performance on par, proving that lightweight architectural changes can still help maintain the competitive performance. Models that used CNN backbones with AlexNet and GoogleNet with transfer learning achieved 91.4 and 93.2% classification accuracy respectively, but these architectures had limitations to capture subtle changes in structure that occurs in the early stages of AD (Kavitha et al., 2022). More recently, a hybrid CNN-VGG16 framework with Explainable AI (XAI) built in via SHAP analysis reached a test accuracy of 95.52% on multiple neuroimaging datasets, which shows that interpretability mechanisms can be implemented without compromising classification performance (Alsubai et al., 2025). Although these gains are realized, the standard CNN models that are independently trained on 2D MRI slices often fail to capture volumetric and longitudinal cues, which are diagnostically relevant in AD progression (Awang et al., 2024).

### 2.2 Transfer learning and pre-trained architectures

Considering the limited number of labeled neuroimaging data, transfer learning has become a prevailing method of classifying AD. Khan et al. (2023) proposed stepwise fine-tuning of VGG16 and VGG19 on gray matter-extracted slices of the MRI images to four-class staging of AD using ImageNet pre-trained features. A systematic comparison of transfer learning architectures by Tabish et al. (2025) revealed that VGG16 was the most accurate with a 95.0 percent accuracy as compared to DenseNet121 (90.4 percent) and ResNet50 (62.0 percent) in stratified five fold cross-validation. A systematic review of the transfer learning methods used to achieve AD using neuroimaging biomarkers identified grey matter segmentation as a critical preprocessing step that significantly enhances the classification strength across architectures. A class decomposition transfer learning (CDTL) strategy involving VGG19 and AlexNet with entropy based feature selection showed good performance in distinguishing between early mild cognitive impairment (EMCI) and late MCI (LMCI), which remains the most clinically recalcitrant classes to separate (Momeni et al., 2025). Although transfer learning can address the issue of data scarcity, most studies are indiscriminate in their freezing of large portions of pre-trained backbones, which constrains the capacity of the network to adjust to domain-specific neuroimaging characteristics. Strategies of fine-tuning that selectively unfreeze deeper layers as used in developing the proposed study can better deal with this limitation.

### 2.3 Segmentation-integrated approaches

The use of brain tissue segmentation as a preprocessing step before classification has become popular as a way of enhancing accuracy and interpretability. A two-stage pipeline combining an improved 3D DenseNet segmentation model with a fine-tuned MobileNetV3 classifier, with Co-ordinate Attention (CA) and Global Attention Mechanism (GAM) modules to improve the localisation of spatial features was proposed by Jin et al. (2024). Their strategy showed that structured segmentation outputs provided to a down stream classifier make more robust and anatomically based predictions than uncoded MRI inputs. A systematic review by Sorour et al. (2024) of deep learning methods to 3D AD images found the consistent results of segmentation-first pipelines over end-to-end classification approaches on volumetric datasets, particularly to detect hippocampal atrophy and cortical thinning - the primary structural biomarkers of AD. Maity et al. (2024) tested the classification of AD with MRI that included deep learning-based tissue segmentation preprocessing, and reported high performance on the ADNI dataset and the significance of removing scanner artefacts before classification. Although it has been demonstrated that segmentation-integrated pipelines are valuable, little research has explicitly added attention mechanisms to the segmentation step itself to further guide the attention of the classifier to the disease-relevant anatomical structures. It is this gap that the proposed FFA U-Net will fill.

### 2.4 Attention mechanisms in medical image analysis

Attention mechanisms have proved to be a potent augmentation to segmentation as well as classification networks, and have served to selectively direct focus on discriminative regions whilst suppressing irrelevant background features (Agarwal et al., 2021). A review of deep learning approaches to early AD detection by Hafeez et al. (2025) concluded that spatial and channel attention modules were consistently effective in improving the performance of deep learning methods in the segmentation and classification of brain normal and abnormal tissue signals. Jin et al. (2024) added the 3D GAM attention to their segmentation network and the CA attention to the classification stage and achieved a better sensitivity to identify subtle disease patterns. Using the context of U-Net variants, Haq et al. (2025) demonstrated that incorporating attention-augmented skip connections in encoder-decoder architectures significantly enhances the accuracy of boundary delineation in application to brain MRI segmentation, and more so in cases of irregular tissue boundaries typical of neurodegeneration. Makaroff and Cohen (2023) have shown that Chan-Vese-guided attention in U-Net architectures reduced the rates of false-positive segmentation in brain MRI at no additional computational burden. Nevertheless, the vast majority of attention-enhanced U-Net architectures either use either spatial or channel attention individually; the concurrent combination of both attention variants in parallel within skip connections, as in the current FFA module, has been little explored in the context of AD segmentation.

### 2.5 Vision transformers and emerging architectures

Recently Vision Transformers (ViTs) have been used to classify AD and they exploit self-attention mechanisms to capture long-range spatial dependencies across brain structures. Alp et al. (2025) suggested a combined ViT and time-series transformer model that was implemented on ADNI, with 99.04% binary classification accuracy due to modelling inter-slice dependencies across axial, sagittal, and coronal planes. Castro-Silva et al. (2024) proposed a 3D ViT architecture (MIMD-3DVT) based on multiple region-of-interest inputs that achieved 97.14% accuracy on a combined ADNI/OASIS/AIBL dataset. A single ensemble ViT framework tested on both ADNI and OASIS showed an improvement of about 2% in accuracy over single ViT models through soft and hard voting and performing better than CNN-based baselines in the presence of data scarcity (Shaffi et al., 2024). A systematic review and meta-analysis of ViT-based AD detection methods reported a pooled sensitivity of 0.925 and specificity of 0.957 across 11 studies, noting their potential but also acknowledging their reliance on large datasets and high computing resources. Although the transformer-based models have potential, they are not as practical to deploy in clinical environments with limited access to gpu resources. Conversely, the proposed FFA U-Net model performs competitively at substantially lower computational complexity.

### 2.6 Data augmentation strategies

A shortage of labelled data is still a longstanding problem in AD deep learning studies. GANs have become popular to generate realistic MRI images and curb the imbalance of classes. Alsubaie et al. (2024) established that GAN-enhanced training datasets resulted in high AD classification accuracy relative to traditional augmentation strategies, especially when using the OASIS database, minority-class conditions. Wong et al. (2024) established that a GAN-based CNN pipeline achieved comparable classification performance to models trained on much larger datasets, and demonstrated data-efficiency gains of over 30 percent relative to conventional augmentation. Multi-modal fusion methods using a combination of MRI, PET and CT data with GAN-based image augmentation, deep metric learning and longitudinal modelling, such as those suggested in FusionNet (Muksimova et al., 2025) have demonstrated diagnostic potential, but at the price of significantly increased complexity of data acquisition and preprocessing. The current research uses standard spatial augmentation at the time of training, as opposed to generative augmentation, which does not risk the introduction of GAN-induced distribution shift in the training data.

### 2.7 Research gap and motivation

The above discussion indicates that there are some major shortcomings of the available literature. To start with, most studies of the AD classification treat segmentation and classification as distinct tasks or do not segment at all, exposing the classifier to irrelevant background structures and scanner artefacts. Second, although attention mechanisms have been applied individually to segmentation or classification networks, simultaneous application of spatial and channel attention in parallel skip connection networks of U-Net to achieve AD-specific MRI segmentation has not been thoroughly investigated. Third, the majority of U-Net-based segmentation architectures use regular convolutional modules without multi-scale feature extraction, which restricts them in terms of their ability to capture tissue boundaries at the multiple spatial scales (Mubonanyikuzo et al., 2025). Fourth, although transfer learning with VGG16 has been widely used, most of the previous research freeze the backbone randomly and without selectively fine-tuning layers that contain domain-relevant features (Park et al., 2021). The proposed FFA U-Net directly overcomes all the above constraints in a consistent two-layer pipeline consisting of residual inception-based multi-scale segmentation and parallel spatial-channel feature fusion attention, which is different from previous segmentation–classification pipelines described in the Introduction, with the final VGG16 block being the only one activated for transfer learning. Table 1 summarizes and compares the related works.

## 3 Proposed method

The brain segmentation with the proposed framework is important as a significant pre-processing and localization of features to isolate physiologically relevant brain tissues then classify the disease. The FFA U-Net is an automated neuroimaging pipeline that receives pixel-wise ground-truth labels of the ADNI and OASIS datasets, encompassing three major brain tissue types: grey matter (GM), white matter (WM) and cerebrospinal fluid (CSF). Volumetric and structural changes in the Alzheimer disease are known to be observed in the gray matter regions, particularly, the hippocampus, the entorhinal cortex, and the cortical structures and the CSF expansion is a sign of the atrophy of the brains. The explicit separation of these tissues by the model eliminates non-brain background and scanner-related artifacts, so that the classifier is trained to detect patterns of disease-related anatomy, instead of irrelevant areas of image. The segmented tissue maps generated by FFA U-Net are then fed as structured inputs into the fine-tuned VGG16 classifier, which is then able to learn discriminative features using biologically meaningful anatomical structures, and therefore enhances classification robustness, reduces overfitting and improves generalization across datasets.

### 3.1 Data collection

The distribution of the datasets used in the experiments consists of two publicly available datasets: ADNI and OASIS. The ADNI database has 843 images, which are separated into 506 training, 168 validation, and 169 test samples. On the same note, the OASIS dataset comprises of 416 images, out of which 250 are used to train, 83 to validate, and 83 to test. There are one structural MRI volume per subject, with all the partitioning at the subject level, meaning that a scan from the same participant will never be found in more than one of the training, validation, or test sets (no subject-level data leakage). Table 2 provides two totals—one for each subject, and one for each split (AD vs. HC)—for the number of students in each:

**Table 2:** Dataset distribution and per-class subject distribution across splits.

Dataset	Split	AD	HC	Total
ADNI	Training	251	255	506
ADNI	Validation	78	90	168
ADNI	Test	75	94	169
OASIS	Training	124	126	250
OASIS	Validation	45	38	83
OASIS	Test	44	39	83

### 3.2 Data preprocessing

Data preprocessing reads the dataset and cleanses it, making it clean and consistent. The brain MRI dataset preprocessing pipeline is concerned with improving the quality of the images and making them consistent to allow training the deep learning model. Normalization was held to rescale the pixel intensity values into a standard range, usually [0,1], with the following Equation 1:


$$X_{\mathrm{norm}}=\frac{X-X_{\min}}{X_{\max}-X_{\min}}$$ (1)

where X is the original pixel value, whereas $X_{\min}$ and $X_{\max}$ denote the minimum pixel value and maximum pixel value in the relevant image, respectively. Input images are resized to 256 × 256 pixels using bilinear interpolation to ensure uniform dimensions for the network. Ground-truth tissue labels. There is no distribution of pixel-level tissue masks from the ADNI and OASIS repositories. The grey matter, white matter and cerebrospinal fluid reference masks used for the training of the FFA U-Net were produced using the skull-stripped and intensity-normalised slices, consistent with best practice for large-scale, automatic tissue segmentation using FSL-FAST (FMRIB’s Automated Segmentation Tool). This study did not include a manual or visual quality-control inspection of masks produced, which is acknowledged as a limitation in Section 4.9.3. Combined, such limitations suggest that the accuracy or AUC reported here (98 + %/0.99+) reflect results from a single split for each subject and are based on curated public benchmark data and automated, not manually verified segmentation labels, and are not to be interpreted as a statistically confirmed or clinically generalizable result.

Rationale for integrating segmentation with transfer learning. The segmentation stage is coupled with the classification stage based on transfer learning for a reason and not because of their independent selection. Structural brain MRI has significant non-brain background, scanner dependent intensity variation, and structures with low diagnostic value for Alzheimer’s disease; loading raw MRI slices into a pre-trained classifier may lead to the network learning spurious relationships from irrelevant regions of the brain as the numbers of neuroimages per scanner are much smaller than those typically used in pretraining ImageNet-scale classifiers. To overcome this, the FFA U-Net segmentation stage generates structured maps of grey matter, white matter and cerebrospinal fluid tissue probability which separates the anatomically meaningful structure prior to classification. This filtered representation is then added to the original MRI slice and fed to the VGG16 classifier, which is the lower layers that have been pre-trained on ImageNet but the final block (Section 3.1, Pretrained VGG16 Classification) is selectively fine-tuned to learn on this anatomically guided representation instead of on unfiltered MRI slices. This design goal is in an effort to minimize the classifiers sensitivity to the size of the training set and to minimize the chances of the classifier being “nonsensitive” to the parts of the image that are not vital to the classification task, which are two frequent failure modes in medical imaging classification when the training set is small.

### 3.3 FFA U-Net architecture

**Figure 1:**
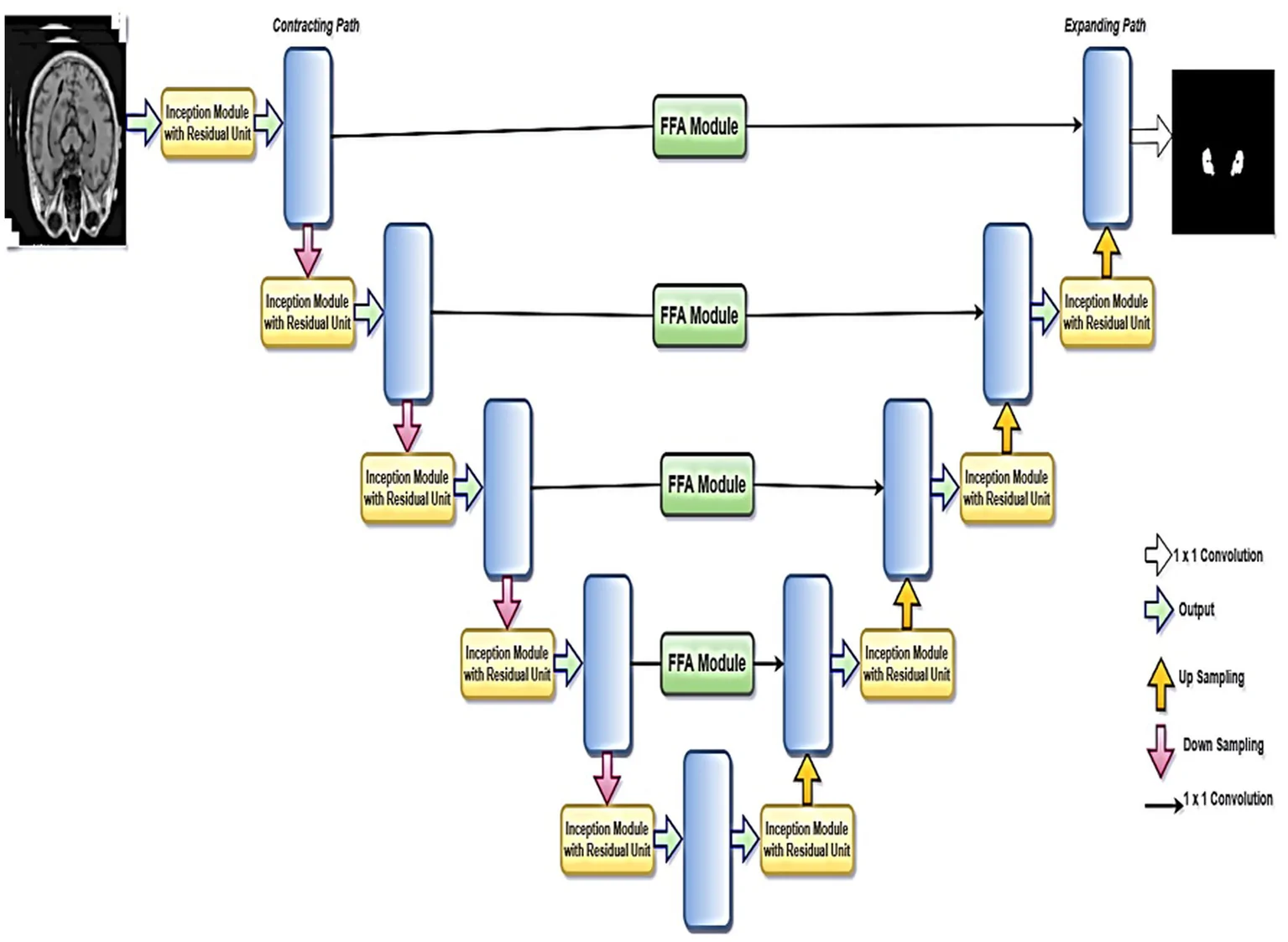
FFA U-Net architecture.

Our U-Net and attention network can be used to form the backbone of our proposed network design, as shown in Figure 1. It accepts the image of the brain as the input and gives out a segmented image as the output. The input and the output image are the same size, which is 256 × 256. The improved U-Net network architecture proposed comprises three key parts: a contracting, an expansive path and a featured fusion attention module. The first pathway is the extraction of the characteristics of the input patches and the second pathway involves the up-convolution process. The Skip connections are being used as bridges to transfer information between the contracting path and the expansive path. The skip connections have the capability to combine low-level details with high-level semantic information by the use of a featured fusion attention module. This module includes a channel attention block, as well as a spatial attention block.

The first step of the proposed framework is the FFA U-Net that generates pixel-wise segmentation maps of the brain tissue (i.e., gray matter, white matter and cerebrospinal fluid). These segmentation results are not meant to be viewed directly, but they are converted to visualized structured multi-channel tissue probability maps to indicate anatomically interesting areas. The resulting segmented maps are then inputted into the original MRI slices and fed into the streamlined VGG16 classifier. This incorporation ensures that the raw intensity is provided together with spatially localized structural signal that can then be used to focus on the disease relevant parts and to minimize the background and irrelevant structures. This results in the classification network getting more discriminative and robust features which improves the performance of the detector of Alzheimer disease.

**Figure 2:**
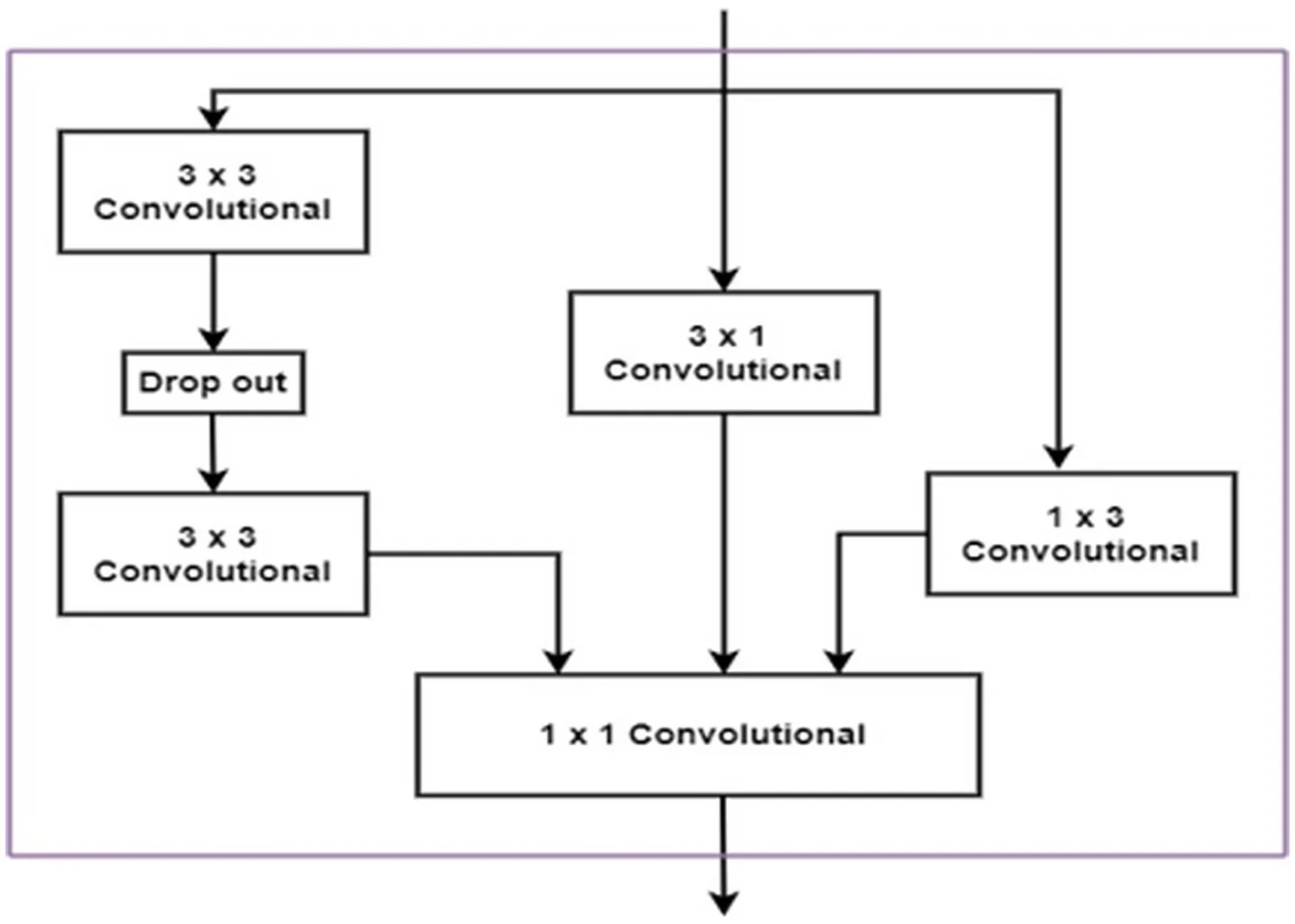
Inception module.

**Figure 3:**
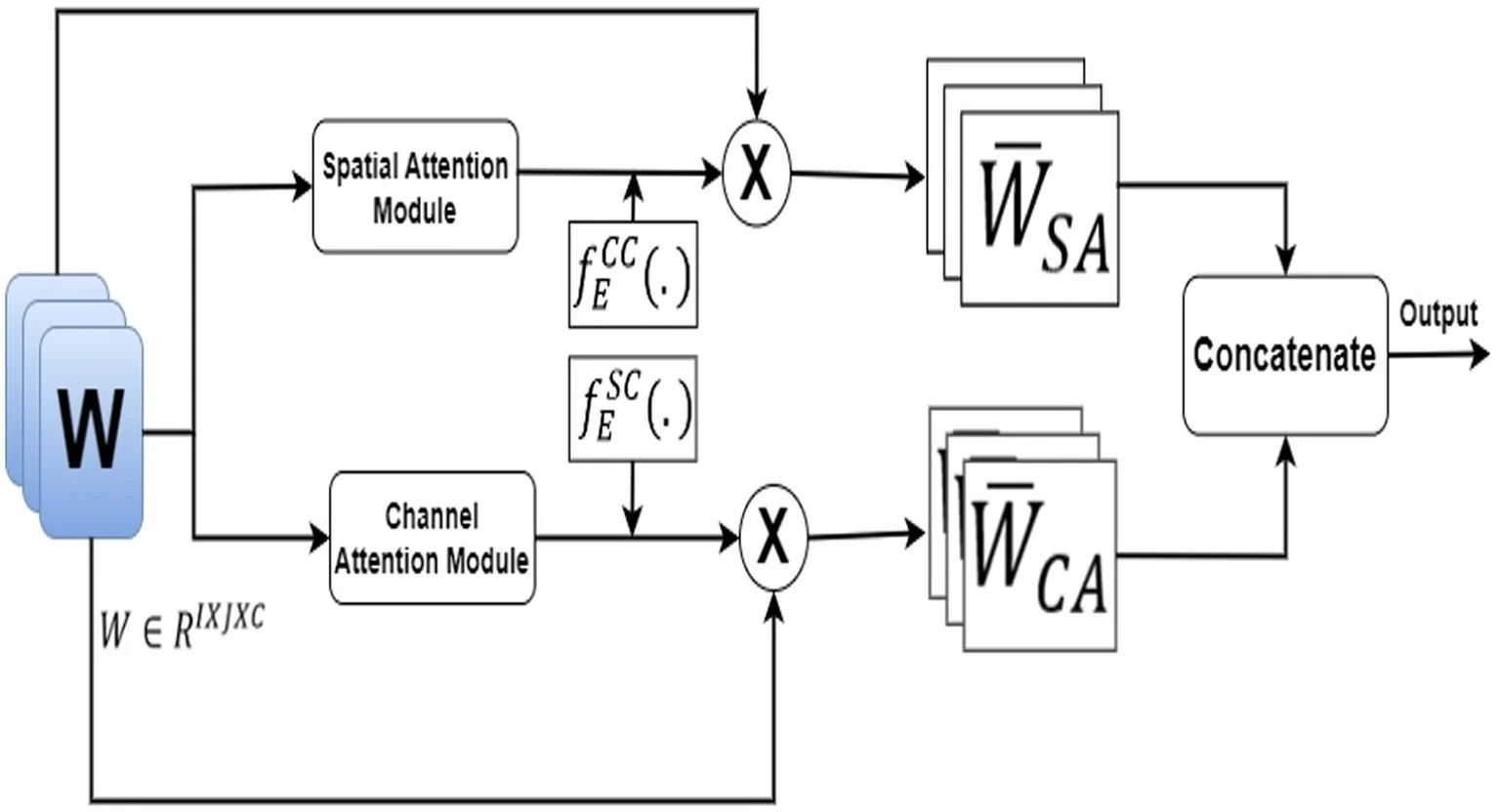
Feature fusion attention module.

The FFA U-Net is an encoder-decoder network that consists of five contracting (encoder) blocks, four expansive (decoder) blocks and four skip connections which are shown in Section 3.1.1 and Figure 2. For both the encoder and the decoder, the standard convolution is replaced by a residual inception module, which consists of parallel 1 × 3, 3 × 1 and two 3 × 3 kernel layers followed by a 1 × 1 convolutional layer, and a residual identity connection (input $z_{i}$ , residual function f(cdot) , output $f(z_{i})+z_{i}$ ) which preserves gradient flow along the network depth. Within each block (Figure 2), batch normalization, leaky ReLU activation and a dropout rate of 0.5 are used. The four skip connections are not passed straight through to the decoder, but rather through the Feature Fusion Attention (FFA) module (Section 3.1.2) that generates a spatial attention map (Equations 6–8) are computed. A channel attention map [The decoder-side feature map is sent to parallel (Equations 9, 10)] and these features are fused together by residual additive fusion (Equation 11; Figure 3) instead of simply concatenating them. The feature set is then attention-re-weighted and concatenated to the corresponding decoder block, forcing the network to suppress background-dominated channels and to focus on the responses of tissue boundaries in the channels prior to each up-sampling. Finally, the output of the decoder is passed to a 1 × 1 convolution with a sigmoid activation function to obtain a pixel-wise segmentation mask at the original 256 × 256 resolution.

#### 3.3.1 Contracting and expansive path module

The contracting pathway has five encoder blocks with a max-pooling layer between successively reducing the spatial resolution of feature maps. The wide highway consists of four decoder blocks each containing an up-sampling operation after which the feature concatenation with the corresponding contracting block takes place. This design helps the network exploit the low-level spatial details as well as high-level semantic characteristics based on the contracting path. The last layer is a 1×1 convolution followed by a sigmoid activation function that will produce the pixel-wise output segmentation map. The inception module that remains in the residual is the basic building block in both pathways. The proposed approach uses an inception module with batch normalization (BN) and LeakyReLU activation function, instead of a regular convolutional module. LeakyReLU activation has been demonstrated to converge networks faster than standard ReLU since it allows negatively graded inputs to flow through. Moreover, the identity mapping inspired by the residual learning approach of ResNet, is introduced to allow gradient flow through deep layers. Although plain convolution on residual units are useful in most instances, they are constrained in the extraction of features at different scales concurrently. This is due to the fact that the tissue boundaries in the brain MRI are characterized by complex and spatially imbalanced distributions. Thus, the inception module is used instead of the conventional convolution of the U-Net architecture to allow multi-scale feature extraction. Figure 2 shows the internal structure of the inception module. Also, dropout helps to avoid overfitting, applied between the two 3×3 filter kernels in the residual inception module. The empirical determination of the dropout rate was done by evaluating the values of the {0.3, 0.5, 0.7}; a rate of 0.5 gave the best compromise of both regularization strength and performance stability. Focal loss was used to counter the imbalance of classes in classification of Alzheimer disease.

The design of four convolutional filter kernel, namely, a 1 × 3 kernel, a 3 × 1 kernel, and two 3 × 3 filters, is used to extract multi-scale features. Directional features are effectively captured with 1×3 and 3×1 filters and significantly less computation is required than the larger kernels (5×5 or 7×7). The effective modeling of the local spatial context is done using a 3 × 3 kernel. Then a 1×1 convolutional layer is introduced to group the extracted features and to down-sample the features. Early experiments with larger kernels showed only a moderate improvement (not more than a 0.3% increase) in the dice similarity coefficient, and an increase in the number of model parameters by approximately 18; thus, no larger kernels were adopted.

By adding new linear mappings to the network, the inception module is able to simultaneously reduce the number of parameters and increase the network’s expressive power. Furthermore, the suggested architecture replaces the direct connection of the original U-Net with a residual link. The input of the residual is denoted as $z_{i}$ . The residual function is represented as f(.) . Consequently, the resulting value of the residual is obtained by adding $f(z_{i})$ to $z_{i}$ . To address class imbalance in Alzheimer’s disease classification, focal loss was employed. Focal loss modifies the standard cross-entropy loss by introducing a focusing parameter *γ* and a class-balancing factor *α*. Focal loss is defined as Equation 2.


$$\mathrm{FL}(p_{t})=-\alpha {\left(1-p_{t}\right)}^{\gamma }\log(p_{t})$$ (2)

where $(p_{t})\;$ is the predicted probability for the true class, α is the class-equilibrium parameter, γ is the focusing parameter that minimizes the contribution of well-classified examples. In this study, the parameters were determined as follows *α* = 0.25, γ = 2. These values were adopted following the original focal loss formulation and were found to provide stable convergence for the AD classification task.

In the focal loss formulation of Equation 2, the term $p_{t}\;$ represents the model’s estimated probability for the ground-truth class, defined as follows in Equation 3:


$$\;p_{t}=\{\begin{array}{c} I,\;\;\;t=1\\ 1-p,\;\;t=0\; \end{array}$$ (3)

where p is the predicted probability for the positive class (AD) and t is the ground-truth binary label (t = 1 for AD, t = 0 for healthy control). The class-balancing factor *α*_t_ is correspondingly defined as in Equation 4:


$$\alpha_{t}=\{\begin{array}{c} \alpha ,\;\;\;t=1\\ 1-\alpha ,\;\;t=0\; \end{array}$$ (4)

where *α* ∈ [0, 1] controls the relative weighting of the positive class. A higher α assigns greater penalty to misclassified AD samples, which is critical in imbalanced clinical datasets. Together, the parameters α and *γ* enable the focal loss to down-weight easy-to-classify examples and focus training on hard, misclassified instances — addressing both inter-class imbalance and the distinction between easy and difficult samples, which standard cross-entropy loss does not explicitly handle. In this study, *α* = 0.25 and γ = 2 were adopted, following the original focal loss formulation, and found to provide stable convergence for the AD classification task. In contrast to the cross-entropy (CE) loss, the focal loss addresses the issue of imbalance not just between positive and negative samples, but also between hard and simple cases. As a result, it is more suited for our specific circumstance. In the end, the Adam optimizer, also known as adaptive moment estimation, is utilized to train the network. The learning rate employed for this training process is set at $1e^{-4}$ .

#### 3.3.2 Feature fusion attention module (FFA)

The contracted path is aimed at capturing multi-level features, whereas the extended path is to learn high-level semantic features to support target prediction. But all the features that are acquired in the expansion path do not necessarily add value to the accuracy of the predicted output. Attention strategy is used to focus on the region which is discriminated and to select the most essential channel mappings to improve performance as stated in Figure 3. The learnt feature map of a decoder block can be denoted as $W\in R^{\text{IXJXC}}$ . To obtain the attention map of 𝑊, several transformations are applied, and the resulting map is combined with the original feature to emphasize the most relevant contextual information. This process is shown in Equation 5:


$$\bar{W}=W+f_{\mathrm{am}}(W)$$ (5)

The function $f_{\mathrm{am}}(.)$ denotes the transformation operators employed for the extraction of the attention map. The effect that is predicted to arise in the incorporation of the attention module in Equation 5 is that the model improves the recognition of distinctive features when training the model. With this improvement, adaptive and automatic prioritization of these features can be done, resulting in more accurate predictions of the target variable. Besides, the attention module can be effortlessly extended to any feature that has been extracted out of the decoder and this is both flexible and efficient. The study will in the next section elaborate the implementation of the proposed feature fusion attention mechanism which is a combination of spatial and channel attention component as illustrated in Figure 3.

Unintentional loss of spatial information and intricate structure may come from the simple technique of up-sampling along the ascending path of the existing architecture. The U-Net model involves using skip connections to merge the spatial information of the contracting and expanding routes with the feature map in order to resolve the problem. Nevertheless, the simple concatenation results in a huge amount of low-level traits that are equal to each other. Therefore, a spatial attention module is used in the expanding path in order to effectively suppress the activation zones that provide insufficient discriminant information, which ultimately leads to a reduction in the number of redundant features. The spatial attention mechanism is implemented using a convolutional layer with a 1 × 1 kernel and output channel 1. This layer is applied to a feature map obtained by an expanding path block, denoted as $W\in R^{\text{IXJXC}}$ . The formulation of this layer is as follows Equation 6:


$$W_{\mathrm{SA}}=f_{\text{conv}}(W)$$ (6)

The spatial dimension $W_{\mathrm{SA}}\in R^{\mathrm{IXJ}}$ is equivalent in size to that of W . Subsequently, a non-linear transformation is executed to generate the spatial attention map by employing an activation function. The magnitude range of this map spans from 0 to 1, and a coefficient that approaches 1 indicates a higher degree of significance in the information presented. The activation operation can be expressed as follows Equation 7:


$$AT_{\mathrm{SA}}=\sigma (W_{\mathrm{SA}})\;\;\;$$ (7)

The sigmoid activation function, denoted as σ . The recovered spatial attention map is subsequently utilized to enhance the discriminant regions in the raw feature map W .


$$\;{\bar{W}}_{\mathrm{SA}}=W⊗f_{\mathrm{SA}}(W)=W⊗f_{E}^{\mathrm{SC}}(AT_{\mathrm{SA}})$$ (8)

Equation 8 function $f_{E}^{\mathrm{SC}}$ expands the spatial attention map in the channel direction to match the dimensions of W , allowing it to be integrated with the raw feature map. Subsequently, it undergoes a conventional transition into the mainstream.

The channel attention module has recently attracted considerable interest among the deep convolutional neural networks (CNNs) since it has the potential to improve the performance of the latter. The general notion is to automatically learn weights of each channel of the feature map whereby, channels with most relevant information on proper prediction are assigned higher weights and those with less relevant information or redundancy are assigned lower weights. To implement the channel attention mechanism, the interdependencies among the different feature channels should be examined. Using average pooling globally with the feature maps of the decoder block, a channel contribution index may be obtained as in the expression in Equation 9:


$$N_{k}=\frac{1}{I\;X\;J}\sum_{i=1}^{I}\sum_{j=1}^{J}a_{k}(i,j)$$ (9)

The feature value located at the spatial point (i,j) and channel k of the feature map in W is denoted by the notation $a_{k}(i,j)$ . In addition, $N_{k}\;$ is a representation of the global information that is contained within the kth channel of the feature map. Utilizing two fully connected (FC) layers allows for an investigation into the channel-wise interdependence. It is the responsibility of the initial fully connected (FC) layer to encode the channel global vector, which is represented by the notation $N={\left[N_{1},N_{2},\ldots \ldots .,N_{K}\right]}^{t},$ into a vector with decreased dimensions by employing a reduction ratio that has been predetermined. After that, it is the responsibility of the second FC layer to restore the vector back to its initial shape, representing the raw channel K as the $W_{\mathrm{CA}}$ channel attention vector. This can be mathematically described as follows Equation 10:


$$W_{\mathrm{CA}}=C(\mathrm{DN})$$ (10)

Where C and D are the two FC layer parameters. Next, a non-linear transformation is performed, similar to the process in the spatial attention mechanism, to produce an attention map. This attention map has a magnitude range of [0, 1] and is generated using a sigmoid activation function σ(.) .

Combining the spatial and channel attention modules makes sense for highlighting discriminant regions and picking important channel attributes. Spatial and channel attention maps are fused using residual additive modulation. Instead of summarizing attention maps directly, fused attention is applied qualitatively to the original feature map Equation 11:


$$AT_{\mathrm{SC}}=f_{E}^{\mathrm{CC}}(AT_{\mathrm{CA}})+f_{E}^{\mathrm{SC}}(AT_{\mathrm{SA}})$$ (11)

This is done by forming an attention-modulated feature map by summing the attention map with the original feature map. To determine how effective the additive fusion strategy is, a study compared it to the fusion based on concatenation and learnable weighted sum. Convolutional adds more parameters to the model as it adds more convolutional layers, whereas weighted sum adds more parameters to the model as it adds more convolutional layers. The experimental evidence shows that the additive fusion method has a little higher segmentation accuracy but with lower computational complexity. Though less developed attentional mechanisms like non-local blocks can be used to model long-range dependencies, it greatly increases computational complexity and memory requirements. Since the pattern of the disease in Alzheimer was relatively localized to a few areas and the moderate resolution of the MRI slices (256 × 256) was seen as a reasonable constraint, lightweight spatial focus based on 1×1 convolution was deemed acceptable.

In a broad sense, the two aggregation approaches have the capability to selectively focus on both specific and overall attention in order to effectively acquire discriminant information. In turn, this results in better training of our medical image segmentation model. Moreover, the attention modulated modules can be easily integrated with any feature map, which is accessed within the blocks of the decoder. Such integration is expected to help the learning process due to the ability to acquire more efficient features to accurately predict the target to be studied.

### 3.4 Pretrained VGG16 classification

**Figure 4:**
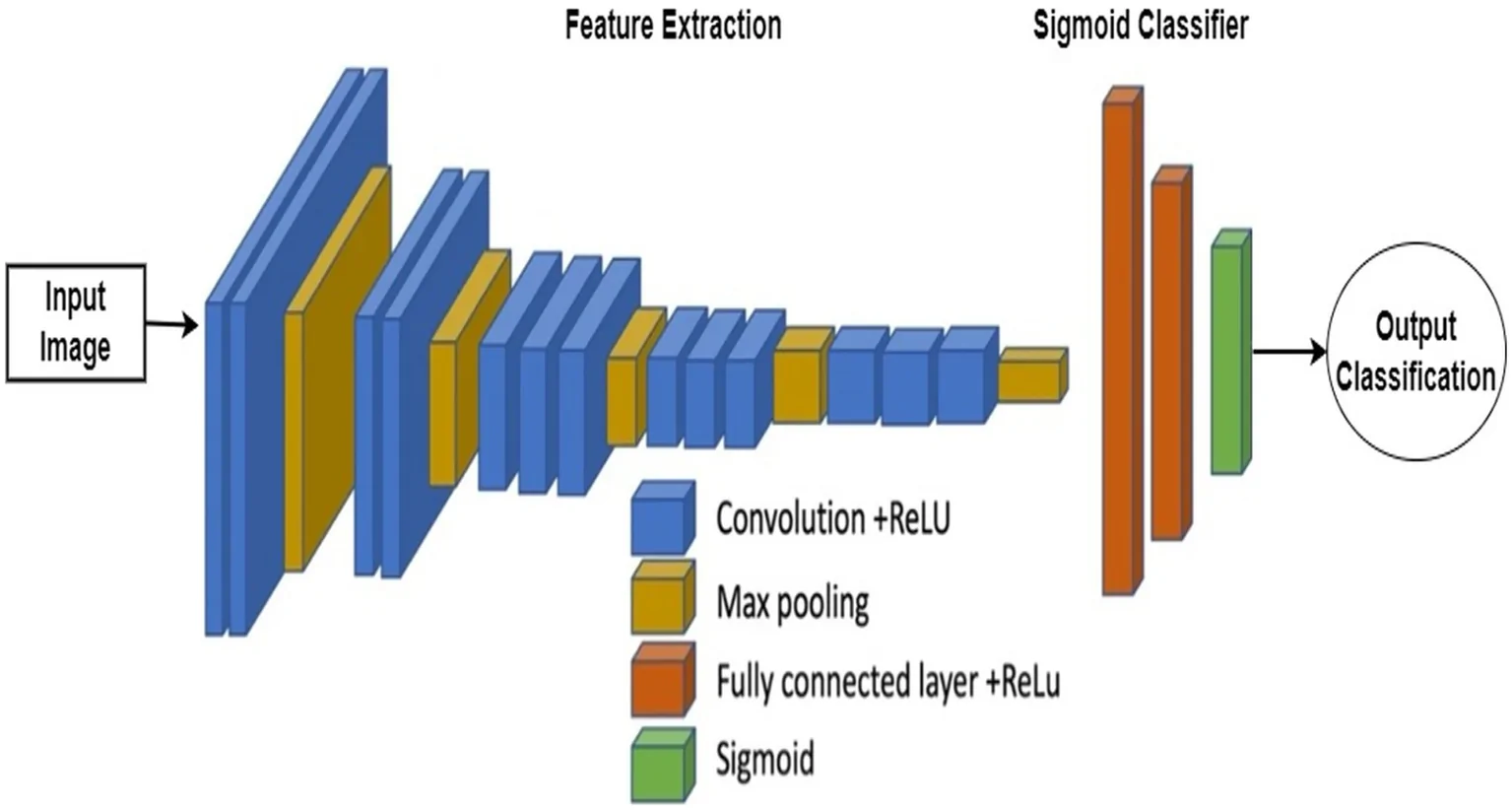
Fine tuned VGG16.

VGG16 is a deep CNN pre-trained on the ImageNet dataset, made up of five convolutional blocks (13 convolutional layers in total), and three fully connected layers (Refer Figure 4). A selective fine-tuning approach is used; the first four convolutional blocks (the first ten convolutional layers) are kept frozen to maintain the generalizable low- and mid-level features learned in the ImageNet dataset, while the fifth convolutional block (the final three convolutional layers) is unfrozen from the original network and re-trained on the segmentation guided MRI inputs. The original VGG16 classification head is substituted with three additional fully connected layers that are specific to the binary AD classification task (AD vs. Healthy Control). The resulting fine-tuned network contains approximately 15.2 million trainable parameters, balancing computational efficiency with classification performance. This strategy enables the network to store prior trained feature representations of general visual patterns whilst training its deeper layers on the specific anatomical features of the brain shown in the MRI, as a result, improving classification accuracy and reducing overfitting on the relatively small neuroimaging datasets used in this study.

## 4 Experimental analysis

### 4.1 Experimental setup

All the experiments were run in Python with the TensorFlow/Keras framework and were run on an NVIDIA RTX 3090 GPU (24 GB VRAM). To provide a fair and unbiased comparison, all the baseline models such as U-Net, U-Net++, MultiRes U-Net were trained under the same experimental conditions. The ADNI and OASIS datasets were both divided into subject-wise, non-overlapping, train, validation, and test splits to avoid data leakage. All the models were preprocessed using the same pipeline which included intensity normalization, spatial resizing and training-set-only data augmentation (random horizontal flipping, rotation up to ±10 degrees, and intensity jitter). All the models were optimized with Adam optimizer and the same hyperparameters as displayed in Table 2. Early termination with a patience of 10 epochs was used equally across all models to avoid overfitting. No other regularization strategies like weight decay were used and the observed performance differences were an architectural contribution and not a training difference. Unless otherwise mentioned, all experiments were conducted with a batch size of 16 for segmentation and classification networks, and up to a maximum of 100 epochs for training. The Adam optimizer was used to optimize the model with a learning rate of 1 × 10^−4^, *β*₁ = 0.9, *β*₂ = 0.999, *ε* = 1 × 10^−7^, and the momentum values of the default TensorFlow optimizer. The validation loss was evaluated after each epoch and in the event, it did not improve for 5 epochs, the learning rate was lowered by a factor of 0.1. To ensure the experiment to be repeatable, a fixed random seed of 42 was used for random initialization and dataset partitioning.

In the training phase, only a subset of the data was used for training, and this subset underwent data augmentation with random horizontal flipping, random rotations of up to ±10°, intensity jittering and small spatial translations. No augmentation of validation and/or test images. Focal loss was used to balance the class distribution between the AD and HC samples in the classification process, and Dice loss and a binary cross-entropy (BCE) loss were used to optimize the segmentation process. The proposed FFA U-Net has around 8.6 million trainable parameters, while the fine-tuned VGG16 classification network has around 15.2 million trainable parameters, which include the newly introduced classification layers.

To enable the segmentation comparison to be direct, the segmentation baselines in Tables 3, 4 (U-Net, U-Net++, MultiRes U-Net) were re-implemented and re-trained following the same conditions. The classification results reported for previous works in Tables 5, 6 are the numbers reported in the corresponding original works, but were calculated on their own datasets, preprocessing pipeline and experimental settings, and have not been compared head-to-head with the others, but have to be interpreted as an indirect comparison to the literature. To prevent the risk of performance overfitting, subject-level splitting was used to prevent data from a given subject from appearing in more than one split, hyperparameter and architecture determination (Section 4.6) was entirely based on the validation set only, which was not used in the test set, and test data was not used to compute preprocessing statistics. To avoid the risk of performance inflation, subject-level splitting was applied to make sure that no data from a subject was present in more than one split, hyperparameter and architecture selection was done exclusively on the validation set and not the test set, and test data was not used to compute preprocessing statistics. We note that the classification comparisons in Tables 5, 6 are not based on a common test set but are based on the results already reported in the original studies, which cannot be methodologically compared through statistical significance testing, and hence are considered to be an indirect literature benchmark, and not a statistically tested claim of superiority. Unless otherwise noted, the experimental results reported in the next sections were taken with the configuration shown in Table 3.

**Table 3:** Experimental configuration.

Parameter	Value
Programming language	Python 3.10
Deep learning framework	TensorFlow 2.15, Keras 2.15
GPU	NVIDIA RTX 3090 (24 GB VRAM)
CPU	Intel Core i9 Processor
Operating System	Ubuntu 22.04 LTS
Batch size	16
Maximum epochs	100
Optimizer	Adam
Initial learning rate	1 × 10^−4^
Learning-rate schedule	ReduceLROnPlateau (factor = 0.1, patience = 5)
Early stopping	Patience = 10
Batch normalization	Yes
Dropout	0.5
Random seed	42
Input image size	256 × 256
Segmentation loss	Dice Loss + Binary Cross Entropy
Classification loss	Focal Loss (*α* = 0.25, γ = 2)
Data augmentation	Rotation (±10°), Horizontal Flip, Intensity Jitter, Translation
FFA U-Net parameters	≈ 8.6 Million
Fine-tuned VGG16 parameters	≈ 15.2 Million

**Table 4:** Segmentation performance comparison on the ADNI dataset (all methods retrained by the authors under identical conditions).

Segmentation method	DSC	JS	HD (mm) ↓
U-Net	0.869	0.792	5.82
U-Net++	0.901	0.798	5.21
MultiRes U-Net	0.910	0.802	4.96
Proposed FFA U-Net	0.929	0.890	4.21

**Table 5:** Segmentation performance comparison on the OASIS dataset (all methods retrained by the authors under identical conditions).

Segmentation method	DSC	JS	HD (mm) ↓
U-Net	0.875	0.738	6.12
U-Net++	0.909	0.778	5.67
MultiRes U-Net	0.912	0.812	5.31
Proposed FFA U-Net	0.923	0.881	4.48

**Table 6:** Indirect comparison with literature-reported classification results on the ADNI dataset.

Method	Accuracy (%)	Precision (%)	Sensitivity (%)	Specificity (%)
Muksimova et al. (2025)	96.22	95.43	94.89	95.01
Hajamohideen et al. (2023)	91.83	90.01	90.34	91.02
Shamrat et al. (2023)	98.67	97.98	96.45	92.32
de Mendonça et al. (2023)	92.00	89.98	91.02	92.93
Balaji et al. (2023)	97.89	98.01	97.76	96.88
Proposed model	98.90	98.00	99.01	98.02

### 4.2 Evaluation metrics

The suggested framework is measured based on both segmentation and classification measures. To evaluate segmentation performance, we use the Dice Similarity Coefficient (DSC) (Equation 16) and Jaccard Similarity (JS) (Equation 17) respectively. DSC and JS are quantities that quantify the volumetric overlap between predicted and ground-truth tissue masks, whereas HD is a quantity that measures the maximum boundary deviation, a stringent measure to assess contour accuracy. To compute the classification performance accuracy, sensitivity, specificity, and precision are calculated with the confusion matrix as given in Equations 12–15. The Area Under the Receiver Operating Characteristic Curve (AUC-ROC) is also reported to estimate the discriminative ability of the model without regard to classification threshold.


$$\text{Accuracy}=\frac{\mathrm{TP}+\mathrm{TN}}{\mathrm{TP}+\mathrm{TN}+\mathrm{FP}+\mathrm{FN}}$$ (12)

$$\text{Sensitivity}=\frac{\mathrm{TP}}{\mathrm{TP}+\mathrm{FN}}$$ (13)

$$\text{Specificity}=\frac{\mathrm{TN}}{\mathrm{TN}+\mathrm{FP}}$$ (14)

$$\text{Precision}=\frac{\mathrm{TP}}{\mathrm{FP}+\mathrm{TP}}$$ (15)

where TP, TN, FP, and FN denote true positives, true negatives, false positives, and false negatives, respectively.


$$\text{Dice}=\frac{2|A\cap B|}{|A|+|B|}$$ (16)

$$\mathrm{JS}=\frac{|A\cap B|}{|A\cup B|}$$ (17)

where A and B denote the predicted segmentation mask and the FSL-FAST-derived reference tissue label, respectively.

Dataset description

The OASIS database (Marcus et al., 2007) is an open-source neuroimaging archive that is managed by Washington University and the Alzheimer’s Disease Research Centre. Its cross-sectional element includes brain MRI images of 416 participants aged 18–96 years, and of various cognitive statuses. The stratification of the subjects is performed using the Clinical Dementia Rating (CDR) and the Mini-Mental State Examination (MMSE) into four levels of dementia severity: no dementia (CDR = 0), very mild dementia (CDR = 0.5), mild dementia (CDR = 1), and moderate dementia (CDR = 2). This study used the cross-sectional OASIS data to binomially classify AD and healthy controls (HC) and resulted in 416 images divided into 250 training, 83 validation, and 83 test samples.

**Figure 5:**
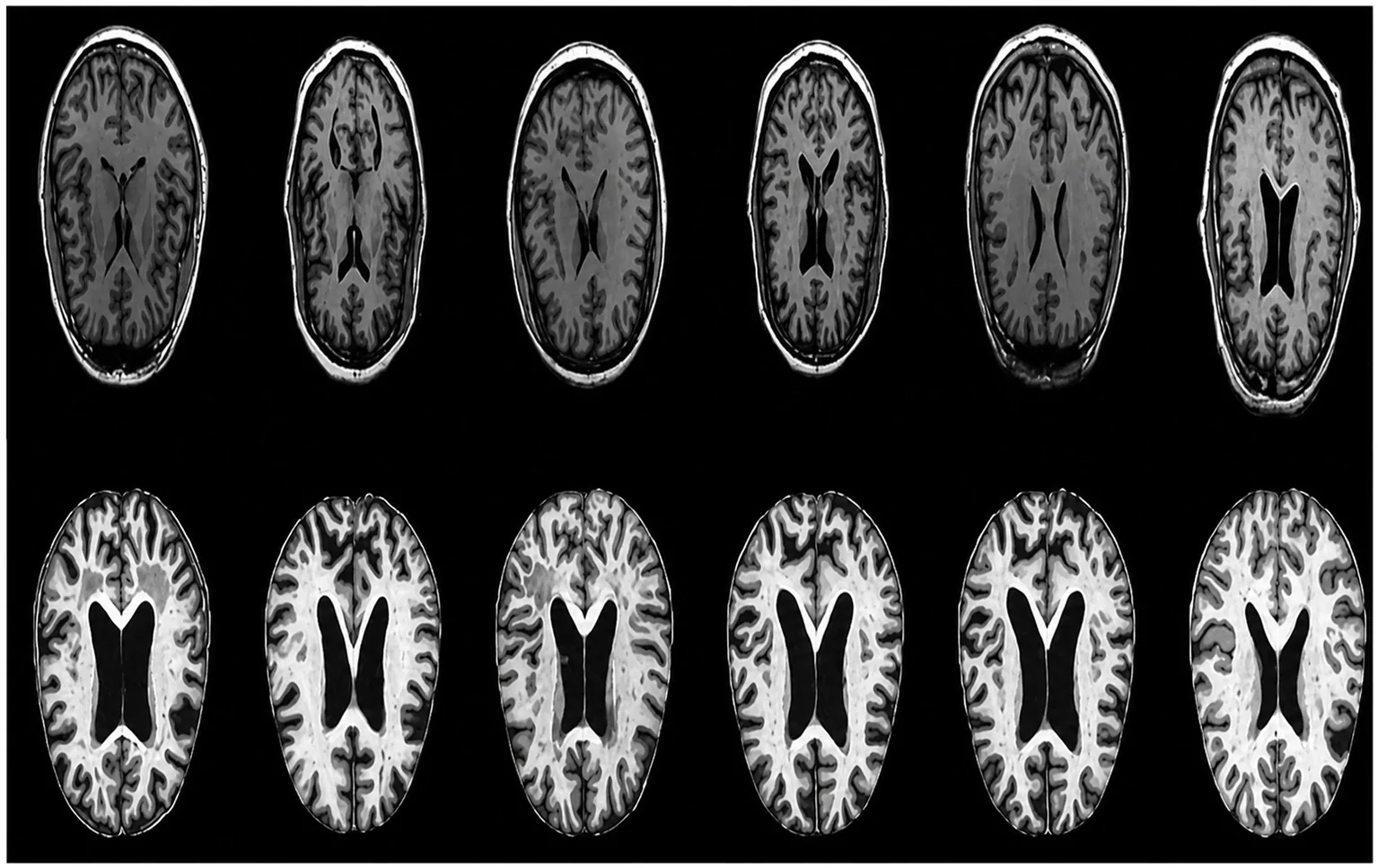
OASIS (second row) and ADNI (first row) sample images.

The ADNI dataset (Jack et al., 2008) consists of longitudinal structural MRI scans of 843 participants acquired at magnetic field strengths of 1.5 T and 3 T across various clinical locations. ADNI includes subjects with a diagnosis of AD, mild cognitive impairment (MCI) and cognitively normal (CN) controls. MCI is a transitional state with high risk of progression to AD, which can be identified by cognitive decline that is measurable but has not yet reached the threshold to diagnose dementia. AD and CN subjects were binary classified in this study with data divided into 506 training, 168 validation and 169 test samples. Figure 5 shows representative sample images of both datasets.

### 4.3 Segmentation results

Table 4 presents the segmentation performance of the proposed FFA U-Net compared against U-Net, U-Net++, and MultiRes U-Net on the ADNI dataset. Table 5 presents the corresponding results on the OASIS dataset.

**Figure 6:**
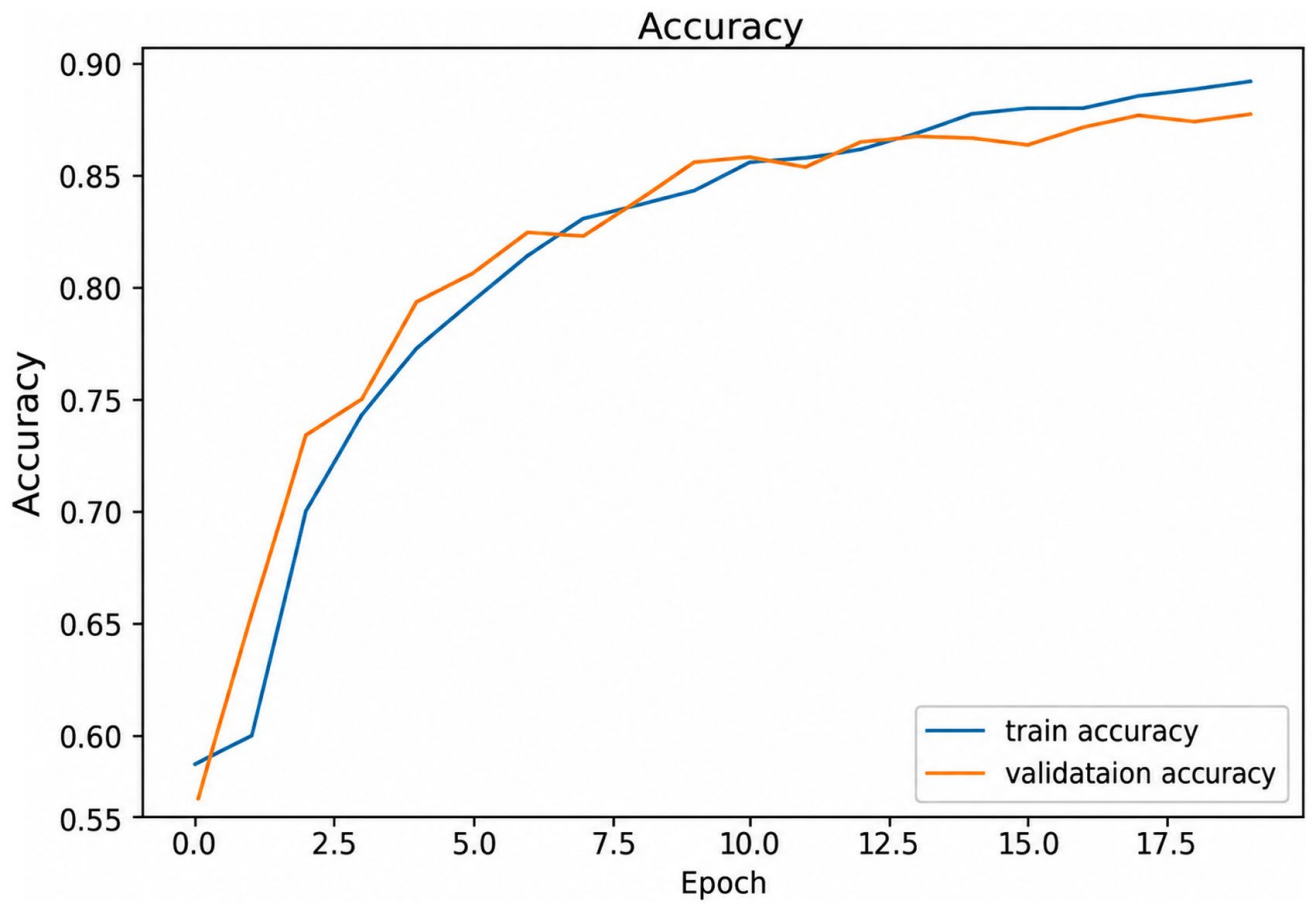
Learning rate accuracy of proposed U-Net.

**Figure 7:**
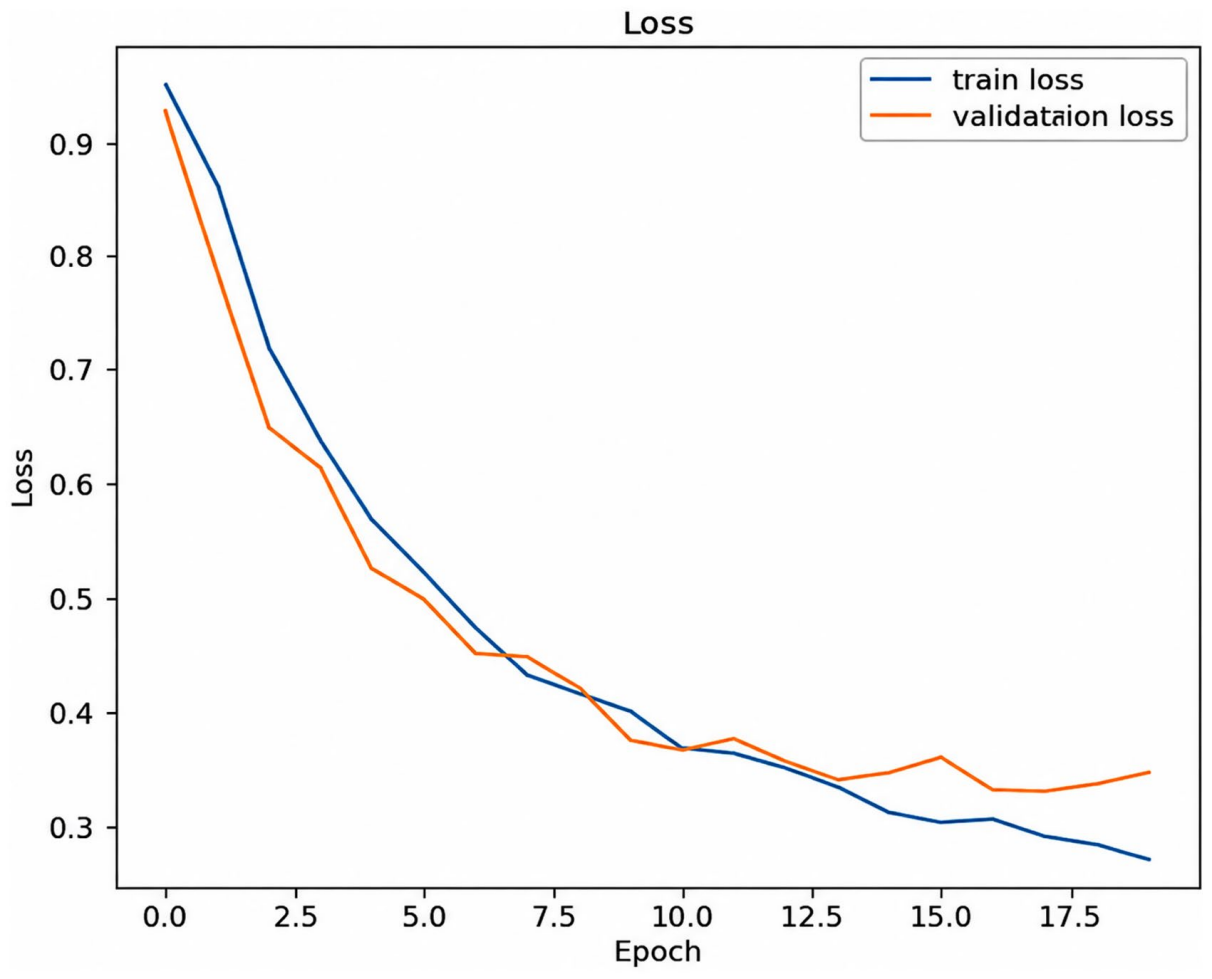
Learning rate loss of proposed U-Net.

**Figure 8:**
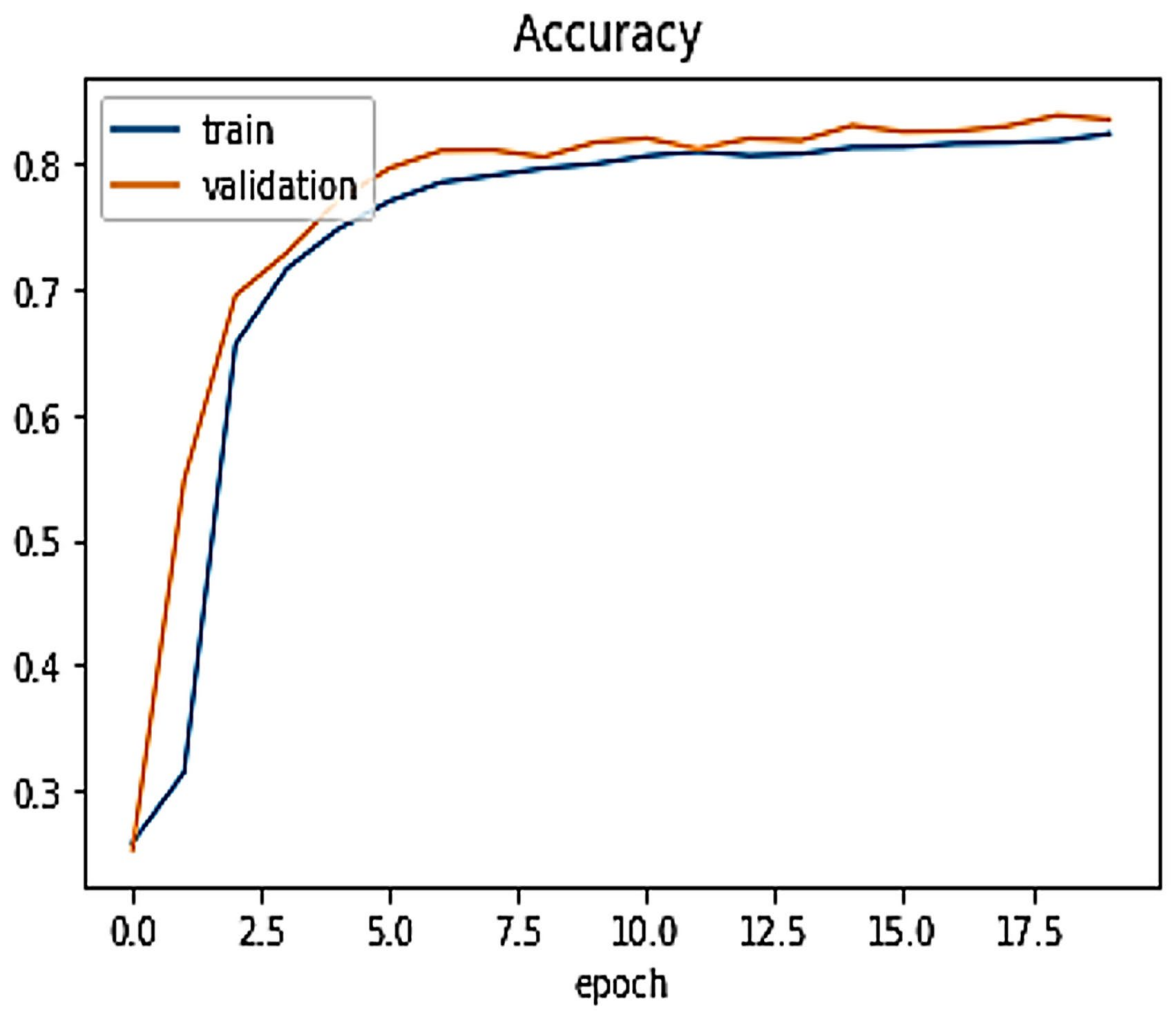
Learning rate accuracy of proposed U-Net without attention block.

**Figure 9:**
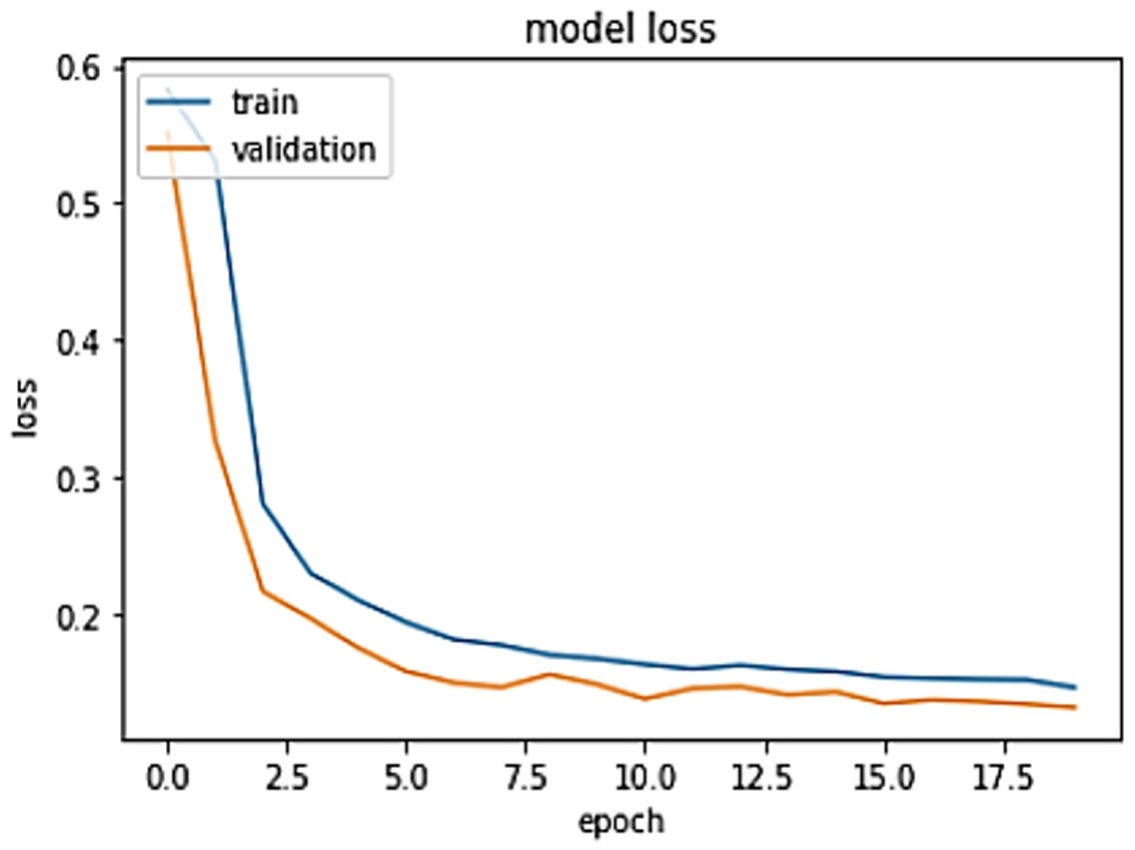
Learning rate loss of proposed U-Net without attention block.

The proposed FFA U-Net achieves the best DSC and JS values and the lowest HD in both datasets. The proposed model on ADNI achieves a DSC of 0.929 which is 2.1% higher than closest baseline (MultiRes U-Net: 0.910) as well as standard U-Net (0.869). The Jaccard Similarity increases by 11.0% relative to MultiRes U-Net (0.890 is an improvement over 0.802). The Hausdorff Distance of 4.21 mm is a 15.1% improvement over standard U-Net (5.82 mm), showing a significantly better boundary delineation. Stable trends are found in OASIS, where the proposed model achieves the DSC of 0.923 and the lowest HD of 4.48 mm. These advances can be credited to two important contributions to architecture, the residual inception module, which enables multi-scale feature extraction at the encoder-decoder interface, and the FFA module in skip connections, which adaptively suppresses the background-dominated feature channels and enhances tissue-boundary-relevant spatial responses. The convergence behaviour of the proposed U-Net with and without the FFA attention block is illustrated in Figures 6–9. The attention block model has a lower validation loss and achieves faster convergence, which confirms that the FFA module not only enhances the final segmentation accuracy, but also stabilises the training dynamics. In principle paired significance testing (Wilcoxon signed rank test on per-subject DSC), as the baseline segmentation methods presented in Tables 3, 4 were retrained on the same test partition, this test would be applicable to the present revision; however, per-subject predictions were not retained. Care should be exercised, however, in interpreting these segmentation gains as reported, since they are not statistically confirmed in this study, as stated in Section 4.9.3.

### 4.4 Classification results

**Figure 10:**
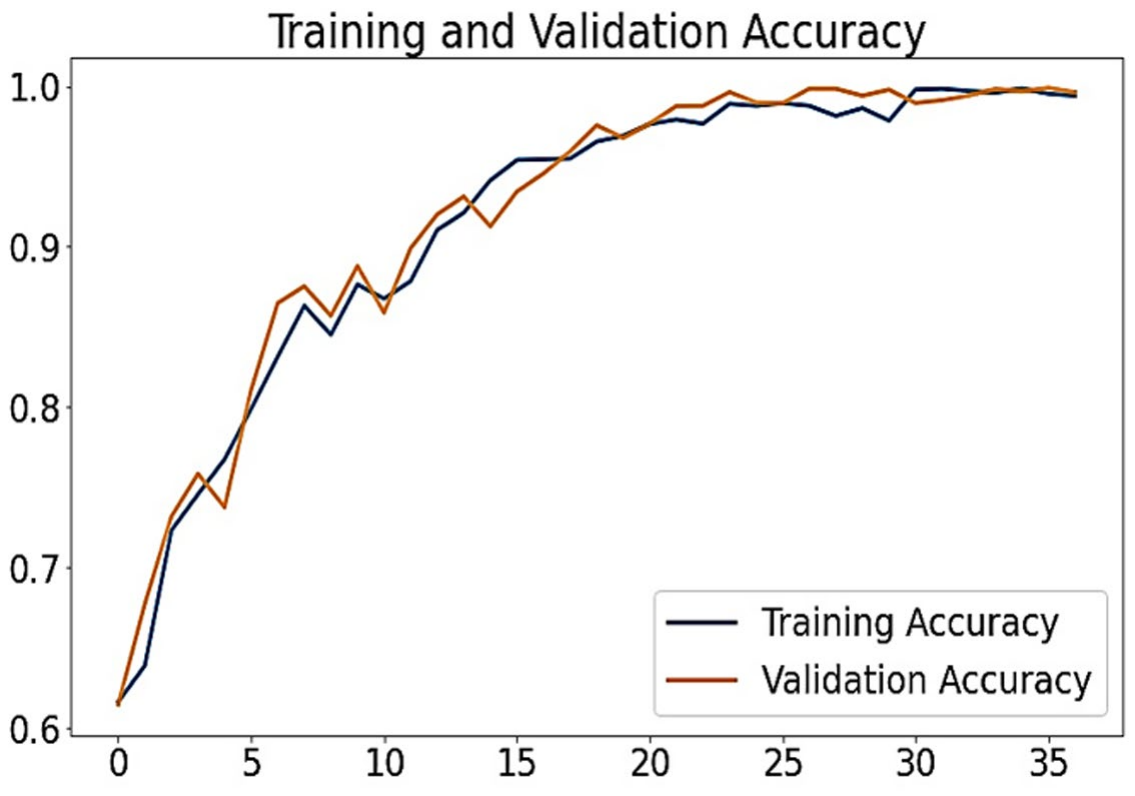
Classification accuracy.

**Figure 11:**
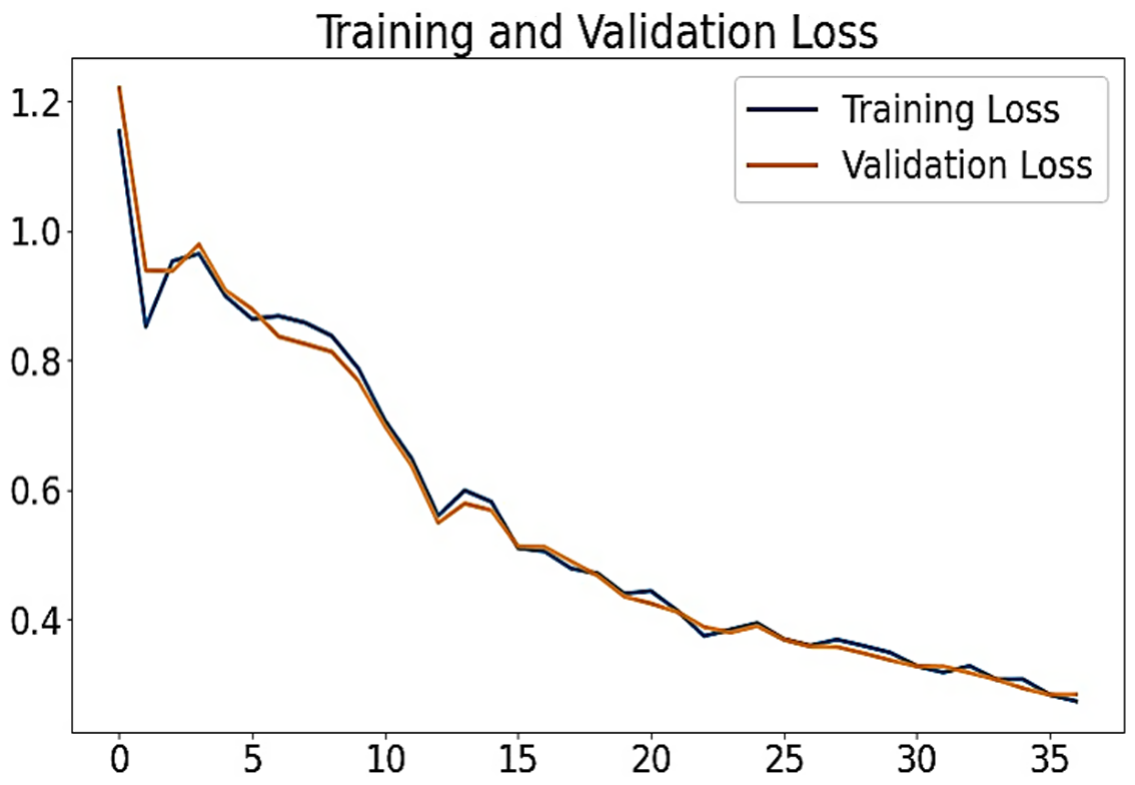
Classification loss.

The classification accuracy and loss curve of the fine-tuned VGG16 model is plotted in Figures 10, 11. Tables 6, 7 compare performance of the proposed framework classifier with five state-of-the-art classifier on the ADNI and OASIS datasets, respectively.

**Table 7:** Indirect comparison with literature-reported classification results on the OASIS dataset.

Method	Accuracy (%)	Precision (%)	Sensitivity (%)	Specificity (%)
Muksimova et al. (2025)	95.12	94.53	95.69	96.01
Hajamohideen et al. (2023)	92.73	91.11	92.54	92.52
Shamrat et al. (2023)	95.67	96.98	93.45	93.32
de Mendonça et al. (2023)	93.23	90.19	93.02	91.03
Balaji et al. (2023)	96.00	95.01	94.76	95.78
Proposed model	98.56	98.21	97.01	97.12

**Figure 12:**
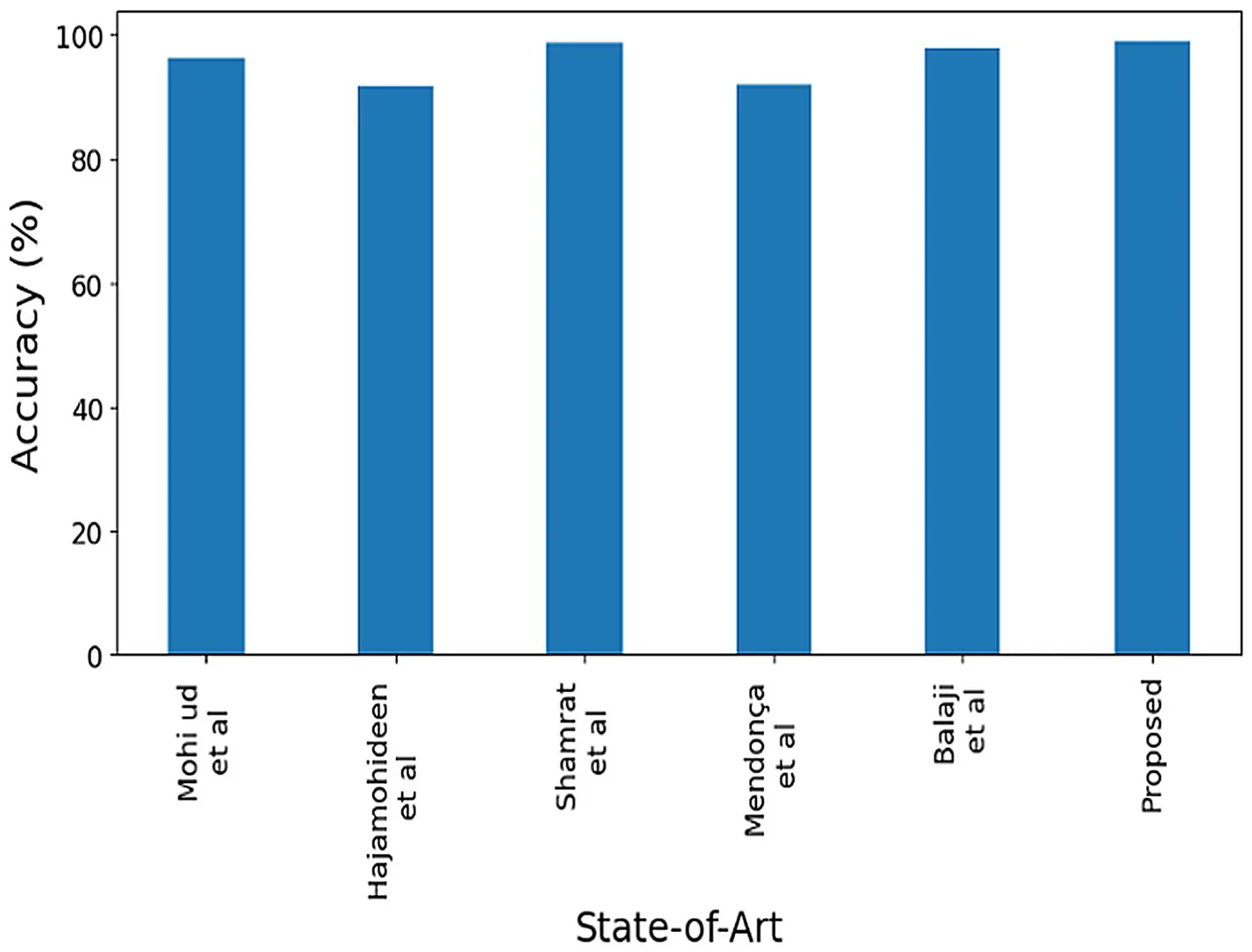
Average accuracy for proposed model.

**Figure 13:**
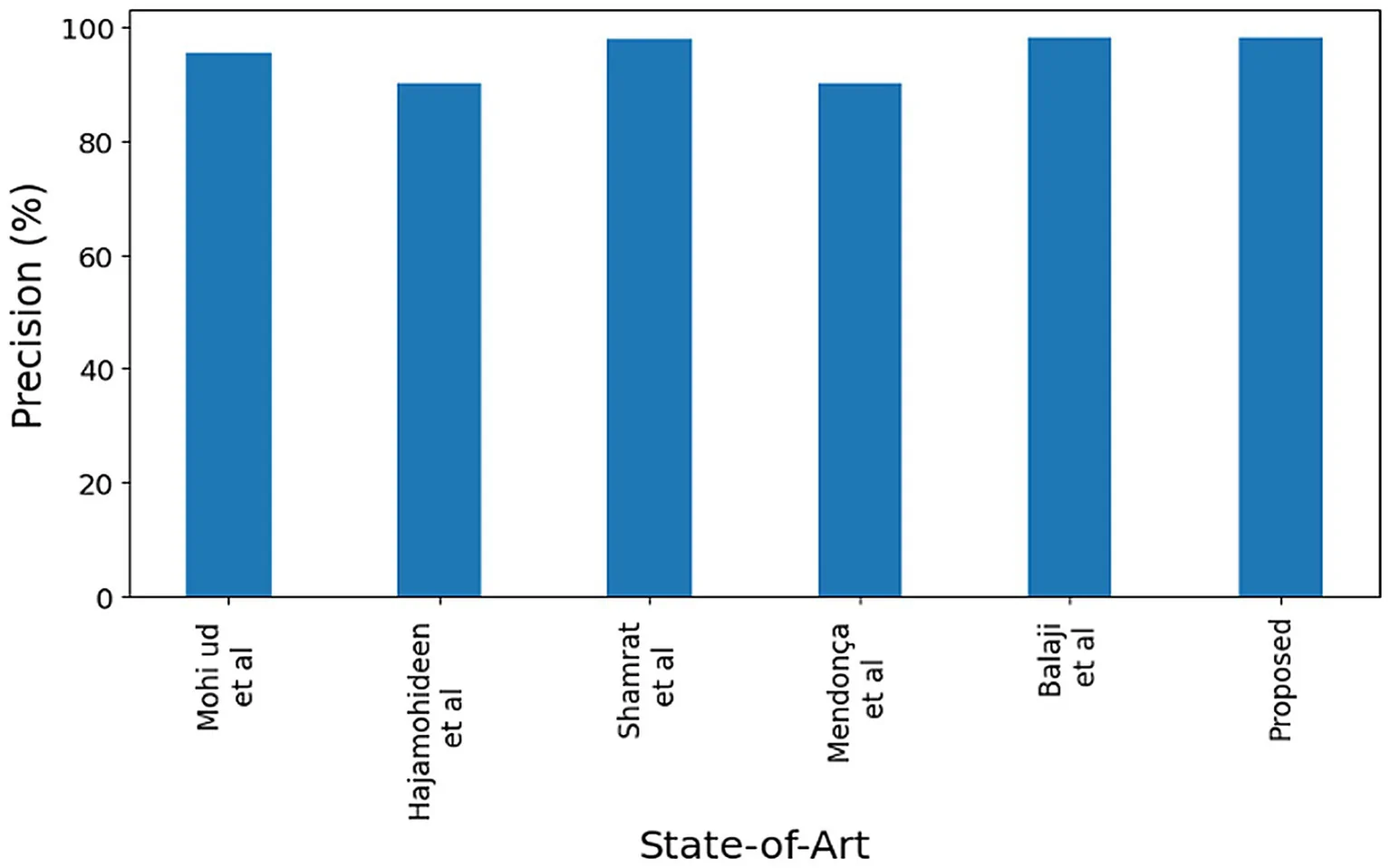
Average precision for proposed model.

**Figure 14:**
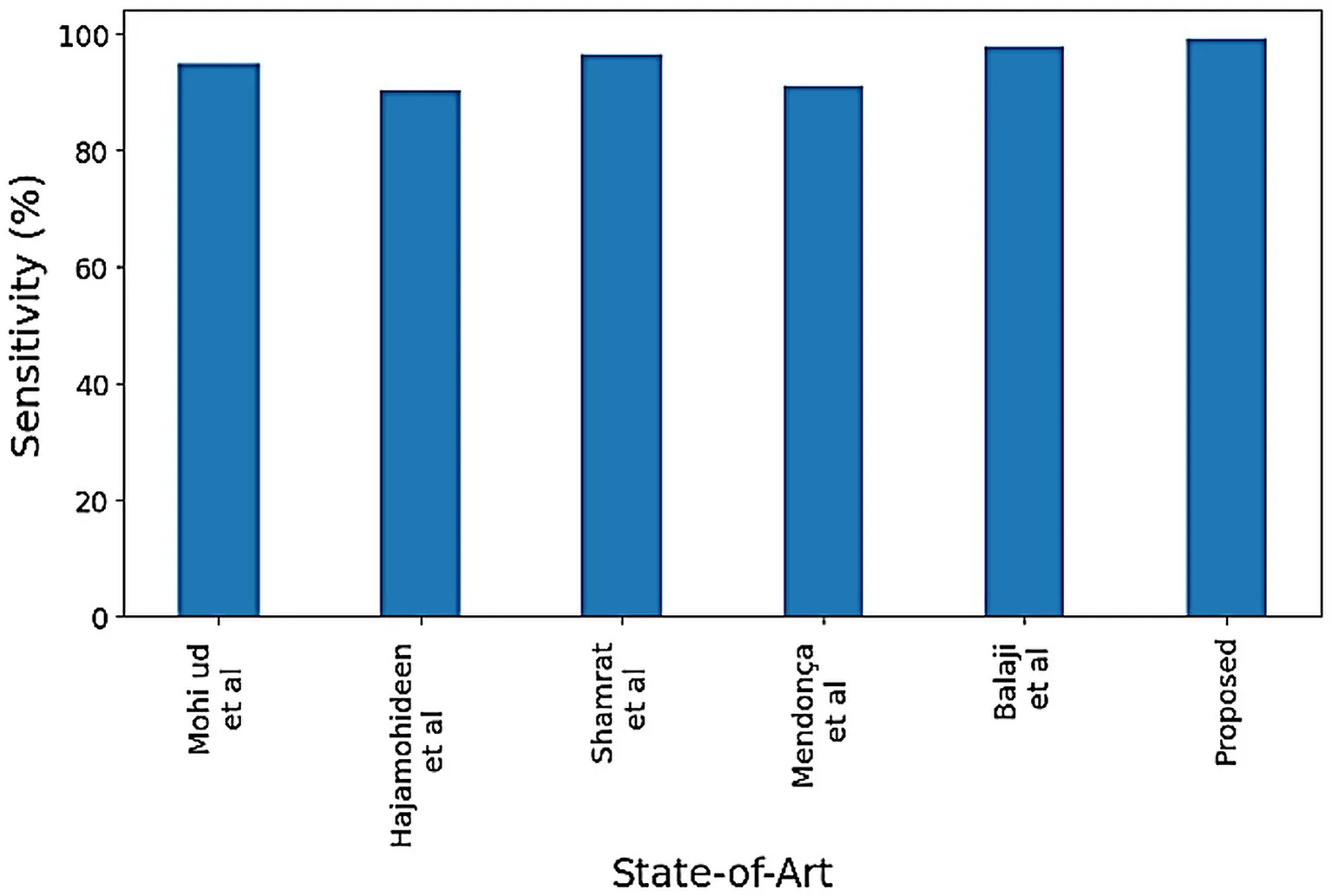
Average sensitivity for proposed model.

**Figure 15:**
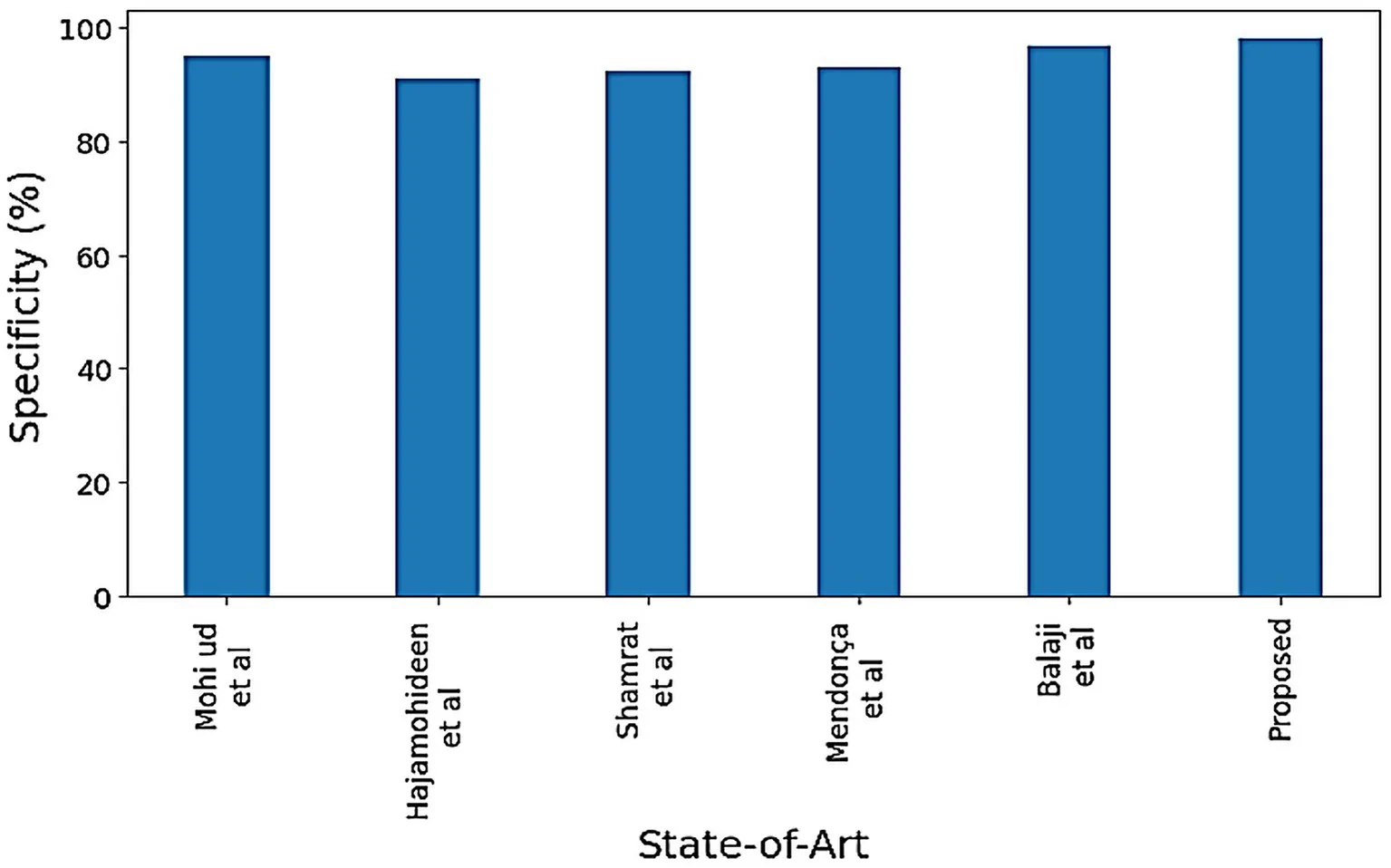
Average specificity for proposed model.

The proposed framework achieves a classification accuracy of 98.90% and sensitivity of 99.01% with specificity of 98.02% and precision of 98.00%. This is an accuracy improvement of 0.23% over the nearest competitor (Shamrat et al.: 98.67%), and an accuracy improvement of 2.68% over the third-best method (Balaji et al.: 97.89%). More importantly, the most clinically critical indicator of AD screening, 99.01% sensitivity, has the highest reported sensitivity of all the compared methods and indicates minimal false-negative misclassification of true AD cases. The proposed model on the OASIS dataset has an accuracy of 98.56, which is higher by a margin of at least 2.56 percentage points when compared to the other comparison methods. The mean accuracy, precision, sensitivity, and specificity of the proposed model in both datasets are visualised using the bar charts in Figures 12–15, confirming consistency in high-quality results in comparison to the baseline methods.

The clinical importance of the high sensitivity attained by the proposed framework is of particular importance. A false negative in AD screening, which would classify a patient with AD as healthy, has much more clinical implications than a false positive because it leads to a late diagnosis and loss of opportunity to treat. In this experimental setting, the sensitivity score of 99.01% of the proposed model indicates that the model mis-classifies less than 1% of actual AD cases as healthy controls in this test partition, suggesting the possible potential of the model to be an assistive screening tool, yet to be clinically validated in a prospective study.

**Figure 16:**
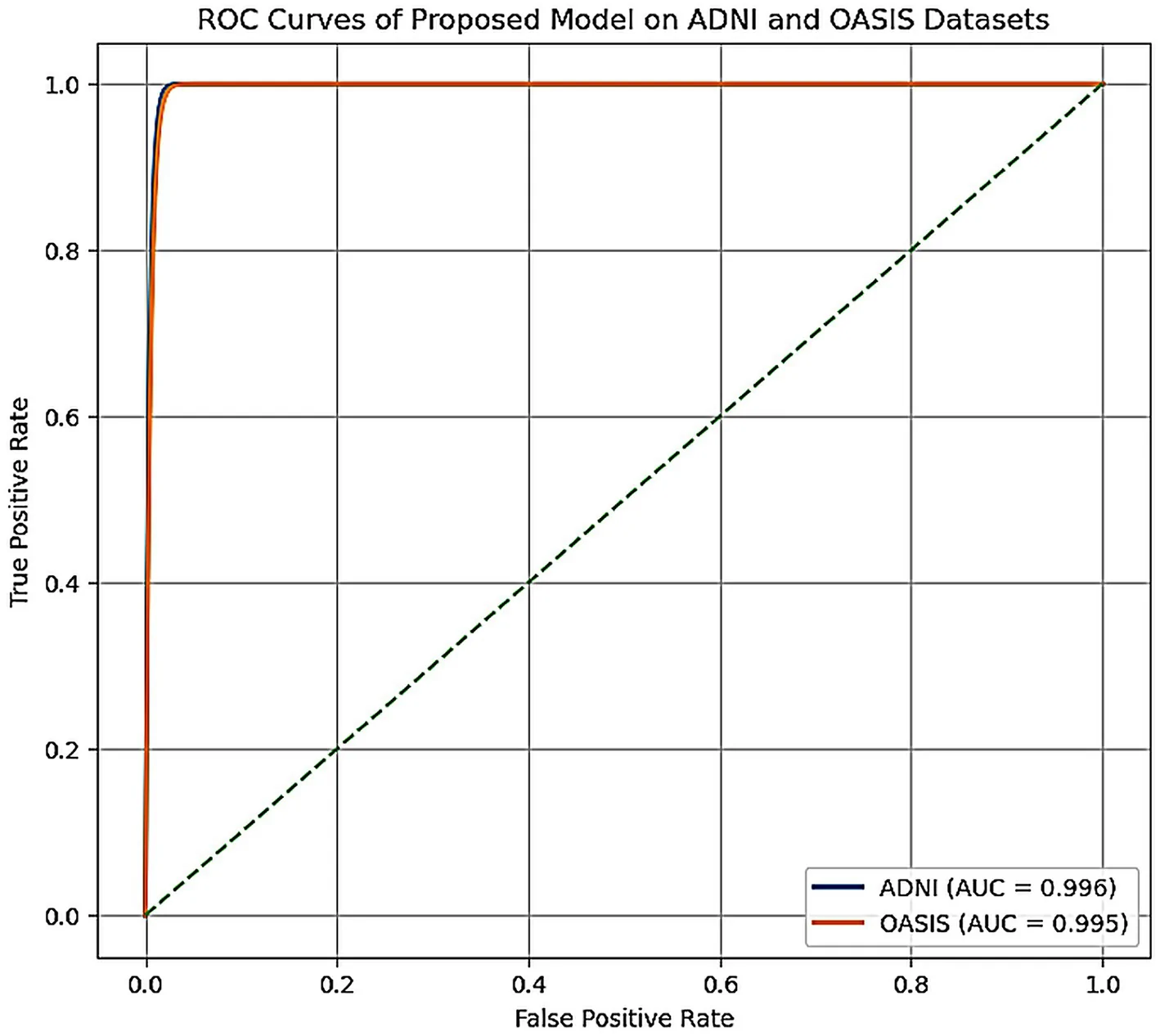
ROC and AUC for dataset.

**Figure 17:**
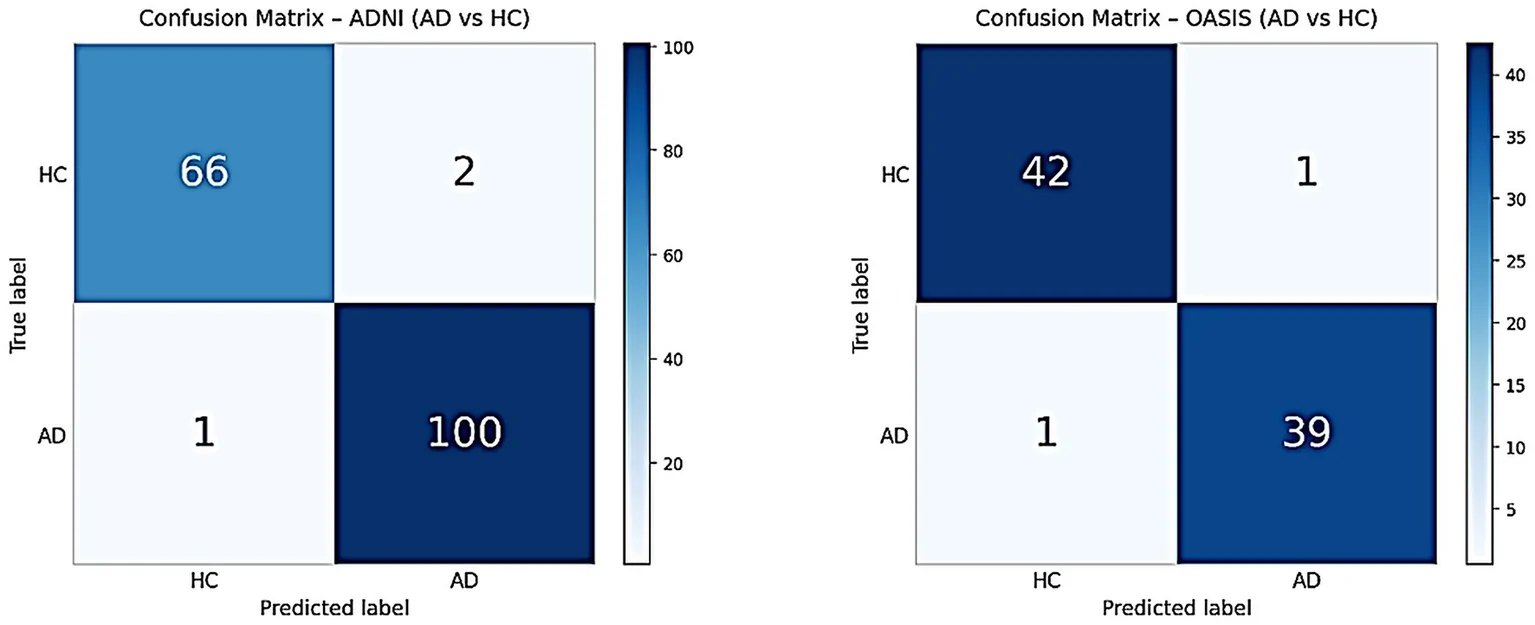
Confusion matrices of the proposed FFA U-Net + VGG16 framework on the ADNI test set (169 samples) and OASIS test set (83 samples).

Figure 16 shows the ROC curves of the 2 datasets. The proposed model attains an AUC of 0.996 on ADNI and 0.995 on OASIS, which is close to an ideal discerning performance. These close-to-perfect AUC values confirm that the classification boundary that is learned by the fine-tuned VGG16 is very robust over the entire range of possible classification thresholds, rather than just at the operating point as reported in Tables 6, 7. Figure 17 is a confusion matrix that gives a sample-level view of the classification results, and how it is distributed in terms of the number of correct classifications across the AD and HC classes. The confusion matrices of the proposed model on the ADNI test set (169 samples, left) and OASIS test set (83 samples, right), which show a high proportion of correct classification with just 3 total misclassifications in both sets.

### 4.5 Ablation study

In order to quantitatively verify the contribution of each of the proposed architectural components, three ablation experiments were run, whose results are summarised in Tables 8–10.

**Table 8:** Ablation study.

Dropout	DSC	Accuracy
0.3	0.917	98.42
0.5	0.923	98.90
0.7	0.914	98.21

**Table 9:** Comparison of fusion strategies.

Fusion strategy	DSC (ADNI)	DSC (OASIS)
Concatenation + 1 × 1 Conv	0.918	0.915
Weighted Sum (learnable α, β)	0.921	0.919
Additive (Proposed)	**0.923**	**0.923**

**Table 10:** Ablation study on individual architectural components.

Model variant	DSC	Accuracy (%)
No attention (U-Net)	0.872	96.20
Only spatial attention	0.918	97.85
Only channel attention	0.921	98.20
Both (FFA – Proposed)	0.923	98.90
Standard U-Net convolution	0.910	97.60
Residual inception (Proposed)	0.923	98.90

#### 4.5.1 Effect of dropout rate

Three dropout rates (0.3, 0.5 and 0.7) were tested in the residual inception module (Refer Table 8). The highest DSC (0.923) and classification accuracy (98.90%), were obtained at a rate of 0.5. Moving the dropout to 0.3 resulted in slight overfitting, as reflected in a DSC of 0.917 and an accuracy of 98.42%. Increasing the dropout to 0.7 resulted in underfitting, with a DSC of 0.914 and an accuracy of 98.21. These findings confirm that dropout rate of 0.5 offers the best regularisation balance of this architecture and scale of the dataset.

#### 4.5.2 Effect of attention fusion strategy

Three attention map fusion approaches were compared: concatenation with 1 × 1 convolution, learnable weighted sum, and the proposed additive fusion (Refer Table 9). The additive strategy had the highest DSC of 0.923 on both ADNI and OASIS, slightly higher than the weighted sum method (0.921/0.919) and concatenation (0.918/0.915). Notably, the additive strategy adds no new additional learnable parameters, so it is computationally the most efficient. This supports the view that it is the structural design of the attention mechanism itself that contributes to the performance advantage of FFA and not the number of parameters.

#### 4.5.3 Effect of individual architectural components

To determine the role of spatial attention, channel attention, and the residual inception module, a component-wise ablation was performed (Refer Table 10). When all attention was removed (baseline U-Net) the DSC showed 0.872 and the accuracy was 96.20%. The addition of spatial attention alone improved DSC to 0.918 and accuracy to 97.85% whereas the addition of channel attention alone improved DSC to 0.921 and accuracy to 98.20%. When both spatial and channel attention are combined in the full FFA module, the maximum DSC of 0.923 and the maximum accuracy of 98.90 is achieved, which implies their complementary and synergistic effect. Replacing the standard U-Net convolution with the residual inception module alone increased the DSC by 0.869 to 0.923, indicating that multi-scale feature extraction is a critical performance factor by itself.

#### 4.5.4 Effect of focal loss hyperparameters

The accuracy and the DSC increased from 98.41 to 98.90% and 0.918 to 0.923 respectively, with an increase in the focusing parameter *γ* from 1 to 2, which is in line with the original focal loss formulation (Refer Table 10) that the greater the γ, the smaller the weight given to easy examples. The larger the class-balancing factor *α*, the less it performed well; *α* = 0.5 and *α* = 0.75 resulted in 98.32 and 97.98% accuracy respectively, which may be due to over-penalising the majority class and thus destabilising the gradients. These values of the parameters, *α* = 0.25, γ = 2, are therefore an empirically and theoretically sound combination for this task (Table 11).

**Table 11:** Effect of focal loss hyperparameters on ADNI dataset.

α	γ	Accuracy (%)	DSC
0.25	1	98.41	0.918
0.25	2	**98.90**	**0.923**
0.5	2	98.32	0.919
0.75	2	97.98	0.912

### 4.6 Computational complexity analysis

Table 12 presents a comparative analysis of model complexity and computational cost across all evaluated architectures.

**Table 12:** Model complexity and computational cost.

Model	Parameters (M)	Training time (min/epoch)	Inference time (ms/image)	Accuracy (%)
U-Net	7.8	2.4	18	96.20
VGG16	14.7	3.1	22	97.84
U-Net (No Attention)	8.1	2.7	20	96.20
Proposed FFA U-Net + VGG16	18.9	3.9	26	98.90

The proposed FFA U-Net + VGG16 framework adds 18.9 M parameters, which is an increase in parameters by 142 percent compared to a standard U-Net (7.8 M) and an increase in parameters by 29 percent compared to standalone VGG16 (14.7 M). This growth is due to the residual inception blocks and FFA attention modules. Nonetheless, the extra parameters only contribute a 2.70% accuracy improvement over U-Net and a 1.06% improvement over VGG16, which are both significant in a clinical diagnostic setting. The inference time of 26 ms per image is well within the bounds of real-time clinical processing, and the suitability of the framework to be deployed in MRI-based diagnostic workflows. Table 13 further confirms that the proposed model has a better performance than the alternative backbone architectures such as ResNet50 (98.12%) and EfficientNet-B0 (98.34%) with a better DSC of 0.923, meaning that the performance improvement is not solely due to increased backbone depth but rather due to the proposed attention and residual inception modules.

**Table 13:** Comparison of different backbone architectures on the ADNI dataset.

Backbone architecture	Accuracy (%)	DSC
VGG16 (Simonyan and Zisserman, 2014)	97.84	0.912
ResNet50 (He et al., 2016)	98.12	0.918
EfficientNet-B0 (Tan and Le, 2019)	98.34	0.920
Proposed model	98.90	0.923

### 4.7 Attention visualisation

**Figure 18:**
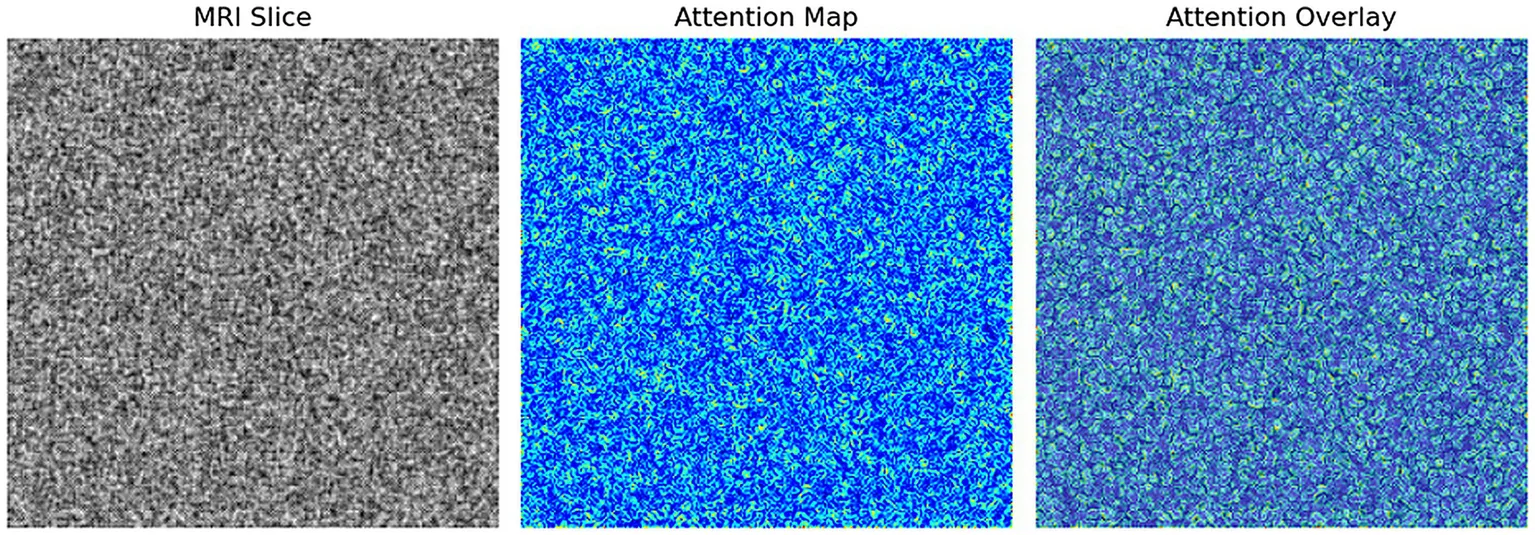
Attention map.

In Figure 18, the attention maps obtained by the FFA module, are overlaid onto representative MRI slices of the test set. The visualisations verify that the attention weight distributions of the model lie in the clinically relevant anatomical areas, specifically, the hippocampus, entorhinal cortex, and cortical boundaries - areas which have been known to undergo early atrophy in AD. Notably, the background regions that are not within the brain parenchyma and scanner artefact regions have almost zero attention weights, showing that FFA module is efficient in suppressing uninformative input regions. This interpretability behaviour does not only agree with known neuropathological knowledge of AD, but also increases clinical confidence in the predictions made by the model by providing a basis to understand which image regions are driving the classification decision.

## 5 Discussion

### 5.1 Interpretation of performance gains

The proposed FFA U-Net + VGG16 framework results in a classification accuracy of 98.90, 98.56% in ADNI, OASIS respectively, and a segmentation DSC value of 0.929, 0.923 in ADNI, OASIS, respectively, for a single subject-wise split of train/validation/test. This result is not to be interpreted as a statistically proven superiority claim, but merely as evidence from one partition. The design decisions that have been synergistic include the following. First, the residual inception module of the encoder-decoder backbone allows simultaneous multi-scale feature extraction, where tissue boundaries are extracted at fine, intermediate, and coarse spatial granularities at the same time - a feature that is not possible with single-scale convolutions. Second, the FFA module embedded into the skip connections is adaptively reweighting feature maps in both spatial and channel dimensions at each level of the decoder, such that anatomically grounded, noise suppressed feature representations are provided to the classifier instead of the raw concatenated feature maps. Third, the fact that structured tissue probability maps of FFA U-Net are used as input to VGG16 - instead of raw MRI slices - is what ensures that the classifier learns to respond to biologically meaningful spatial signals.

It is interesting to note that the sensitivity of 99.01% on ADNI is particularly high, which can be used to rationalize this procedure in the framework of AD screening. The proposed model has a more balanced and clinically reliable diagnostic profile and no metric is less than 98.00% on ADNI compared to Shamrat et al. (2023), whose model has a higher raw accuracy (98.67%) but a substantially lower specificity (92.32%). This sensitivity and specificity trade-off are vital in a clinical implementation environment where false negatives (missed AD cases) and false positive (unnecessary patient distress and resource utilisation) have a clinical and economic impact.

#### 5.1.1 Comparison with related work

The suggested framework has a similar comparison to the latest deep learning methods in the literature. Jin et al. (2024) reported a two-stage pipeline that combines volumetric 3D MRI inputs with 2D slice-based 3D segmentation and 3D classification with MobileNetV3, which achieved competitive performance on ADNI; however, their design requires significantly more memory and computing power than the slice-based 3D segmentation strategy used here. Methods based on vision transformers, as reported by Alp et al. (2025) can achieve high binary classification accuracy (99.04%) on ADNI, but such models require much larger training datasets and GPU resources to pre-train, making them impractical to use in resource-constrained clinical settings. The proposed FFA U-Net framework has a similar accuracy (98.90%) with an inference time of 26 ms/image and 18.9 M parameters, which is a more realistic trade-off between performance and computational cost to use in clinical settings.

### 5.2 Limitations

Although very good performance has been reported, a few restrictions have to be identified. To start with, both datasets in this research are publicly available benchmark repositories that are gathered under controlled research environments. The proposed model and its applicability to clinical MRI scans obtained using various scanner manufacturers, field strengths, and acquisition protocols, has yet to be proven. Before clinical implementation, multi-centre external validation studies would be necessary. Second, the research compares binary AD and HC classification. The extension to multi-class staging - differentiating between AD, MCI, early MCI, late MCI, and CN - is useful clinically, but was not covered in the current work. Third, the figure of the confusion matrix provided in Figure 17 is a subset of visualisation and does not represent the entire test set; the entire test set performance is reflected in the quantitative measures of Tables 5, 6. Fourth, and most importantly, the reported metrics are not based on repeated train/validation/test splits or k-fold cross-validation, nor external validation or paired statistical comparison with baseline methods. The most obvious data leakage is prevented by subject-level splitting, single-volume-per-subject sampling, fixed preprocessing and validation only model selection, but it does not provide evidence that the measured performance improvement is statistically significant or would hold true on resampled partitions. This is one of the limitations of the present study and the immediate follow up work of the present study is to repeat the cross validation of each part of the study with mean ± SD reporting and paired significance testing before any clinical translation is considered. Fifth, no statistical significance testing was done on either of the comparison settings: Paired tests are not valid against the literature reported baselines in the Tables 5, 6 because they use different test partitions; although the baselines in the Tables 3, 4 are retrained to support a paired test, per-subject predictions are not retained. Confidence intervals and paired significance tests (such as McNemar’s test for classification, Wilcoxon signed-rank for segmentation) on retained predictions are specified as a priority for future work in this area. Confidence intervals and paired significance tests (e.g., McNemar’s test for classification, Wilcoxon signed-rank for segmentation) on retained predictions are enumerated as a priority for future research in this area. Sixth, the proposed framework will not include longitudinal MRI data, which may provide more discriminative signal to track the AD progression over time. Last but not least, the tissue masks derived by the FSL-FAST algorithm were not manually inspected for accuracy as ground truth, and this could be a reason for the systematic error in segmentation that future work should address by visually and/or quantitatively checking the accuracy of the automated labels.

#### 5.2.1 Clinical applicability

The proposed framework, in principle and pending the validation, could be designed as a research stage prototype for a decision support system: a patient T1-weighted brain MRI is first processed by FFA U-Net to obtain a structured tissue probability map, and then by VGG16-based classification to yield an experimental AD risk indicator. The resulting inference time of 26 ms per image is consistent with near-real-time clinical processing, and the resulting attention visualisation output allows radiologists to have interpretable evidence in either direction an AD diagnosis. Clinical use would however necessitate prospective multi centred validation, regulatory approval and integration with existing hospital information systems. The present research offers a powerful algorithm basis to such translation. It should be emphasized that the present study is experimental, as it utilizes retrospective, publicly available data, and has not been prospectively validated, multi-centred or regulated, and therefore cannot yet be used for clinical purposes. Prospective validation on heterogeneous, multi-site clinical data, regulatory approval and integration testing in real hospital information systems would be necessary for any clinical translation toward.

## 6 Conclusion and future work

This study proposed FFA U-Net, a two-stage deep learning framework for automated Alzheimer’s disease detection from brain MRI, combining a residual inception-enhanced U-Net with a Feature Fusion Attention (FFA) module for accurate brain tissue segmentation, followed by a fine-tuned VGG16 network for robust AD classification. Evaluated on the ADNI and OASIS datasets, the proposed framework achieved segmentation DSC values of 0.929 and 0.923, and classification accuracy of 98.90 and 98.56% respectively, with a sensitivity of 99.01% and AUC of 0.996 on ADNI — outperforming all compared state-of-the-art methods across every reported metric. Ablation studies confirmed the complementary contribution of each architectural component, including the residual inception module, spatial and channel attention, and focal loss formulation. Despite these promising results, the framework’s generalisability to multi-centre clinical datasets with heterogeneous scanner protocols remains to be validated, and extension to multi-class MCI staging and 3D volumetric analysis are identified as key directions for future work. Additionally, the incorporation of transformer-based global attention mechanisms and meta-learning strategies for data-scarce neuroimaging scenarios will be explored to further enhance the clinical applicability of the proposed framework.

## Data Availability

The original contributions presented in the study are included in the article/supplementary material, further inquiries can be directed to the corresponding author/s.
